# Word Learning by Preschool-Age Children: Differences in Encoding, Re-Encoding, and Consolidation Across Learners During Slow Mapping

**DOI:** 10.1044/2022_JSLHR-21-00530

**Published:** 2022-04-20

**Authors:** Katherine R. Gordon, Stephanie L. Lowry, Nancy B. Ohlmann, Denis Fitzpatrick

**Affiliations:** a Center for Childhood Deafness, Language and Learning, Boys Town National Research Hospital, NE

## Abstract

**Purpose:**

Children with typical development vary in how much experience they need to learn words. This could be due to differences in the amount of information encoded during periods of input, consolidated between periods of input, or both. Our primary purpose is to identify whether encoding, consolidation, or both, drive individual differences in the slow-mapping process.

**Method:**

Four- to 6-year-old children (*N* = 43) were trained on nine form-referent pairs across consecutive days. Children's ability to name referents was assessed at the beginning and end of each session. Word learning was assessed 1 month after training to determine long-term retention.

**Results:**

Children with varying language knowledge and skills differed in their ability to encode words. Specifically, children varied in the number of words learned and the phonological precision of word forms at the end of the initial training session. Children demonstrated similarities in re-encoding in that they refined representations at a similar rate during subsequent sessions. Children did not differ in their ability to consolidate words between sessions, or in their ability to retain words over the 1-month delay.

**Conclusions:**

The amount of experience children need to learn words is primarily driven by the amount of information encoded during the initial experience. When provided with high-quality instruction, children demonstrate a similar ability to consolidate and retain words. Critically, word learning instruction in educational settings must include repeated explicit instruction with the same words to support learning in children with typical development and varying language skills.

**Supplemental Material:**

https://doi.org/10.23641/asha.19606150

Children with typical development (TD) vary substantially in the rate that they add words to their vocabularies during infancy and early childhood (McKean et al., 2015; Taylor et al., 2013). These differences matter. Language skills at Kindergarten entry predict success in a variety of academically relevant areas including language, mathematics, and social skills (Pace et al., 2019). Educators and policymakers have long been interested in factors that affect vocabulary growth during early childhood (Common Core Standards Initiative, 2021). Researchers have identified both external and internal factors related to this growth. External factors include the quantity and quality of language input (Masek et al., 2021; Rowe, 2012) and socioeconomic status (SES; McKean et al., 2015; Taylor et al., 2013). Internal factors include inhibitory control (Hadley et al., 2021; M. M. McClelland et al., 2007), attentional processes (Smith & Yu, 2013), and children's vocabulary knowledge when starting vocabulary instruction (Penno et al., 2002). Understanding these factors is essential to design effective educational practices to optimally prepare children for school entry (Hadley et al., 2021).

Word learning, like any kind of learning, is inextricably tied to memory processes (Wojcik, 2013). Thus, an important internal factor associated with word learning is individual differences in memory processes. To date, researchers have focused primarily on working memory, what children are able to learn about words during a period of input. However, because word knowledge is built across many experiences (Swingley, 2010), a child could demonstrate slower vocabulary growth because she learns less information about words during periods of input, forgets more information about words between periods of input, or both. We review these processes in more detail below. Critically, to understand individual differences in vocabulary growth during early childhood we must understand how cognitive factors, such as memory processes, affect children's performance in dynamic word learning tasks (Samuelson, 2021). It is through understanding the dynamic process of word learning that we can design instruction that is most likely to boost vocabulary growth in a variety of learners.

The Complementary Systems Account provides a model of how memory processes affect word learning (Davis & Gaskell, 2009; J. L. McClelland et al., 1995). According to this account, word learning occurs across two essential stages. The first stage includes rapid learning from input. During an initial experience with a word the child may encode representations of the word form, which includes the phonemes that make up the word and their order, the word meaning, and the link between the two (see Figure 1). These representations are related to hippocampal activity (Dumay & Gaskell, 2007) and this process is referred to as fast mapping (Swingley, 2010). These initial representations are often imprecise (Edwards et al., 2004) and tend to decay rapidly from memory after the input is withdrawn (Munro et al., 2012). However, representations of the form, meaning, and link can be refined and strengthened across multiple exposures within a single learning session (Bishop et al., 2012).

**Figure 1.**
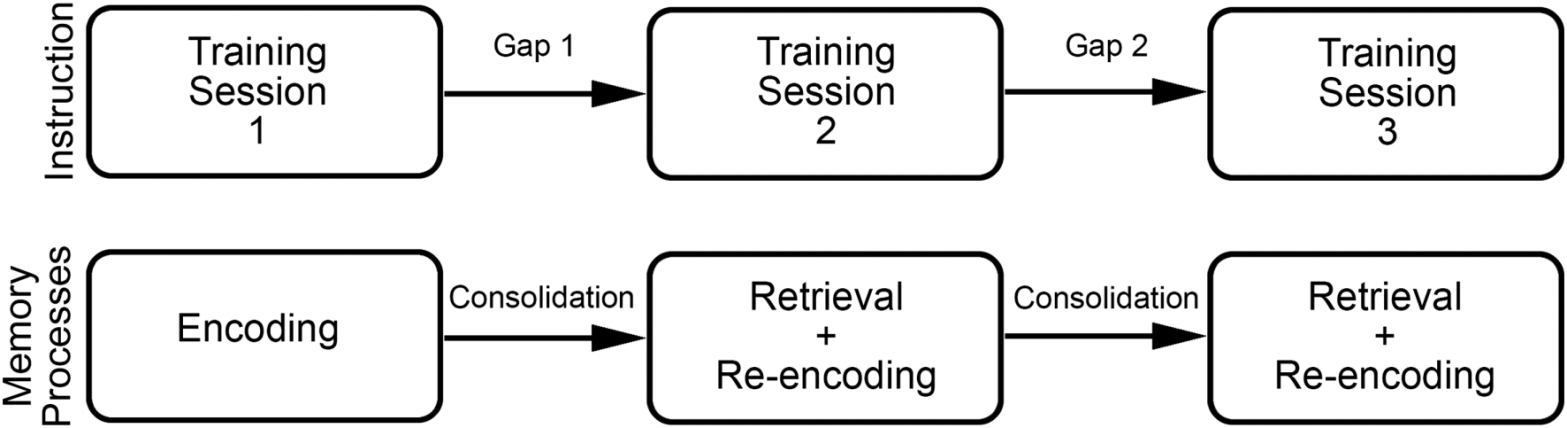
The memory processes involved in slow mapping.

The second stage includes slower consolidation over time. After the initial period of encoding, representations must be consolidated to be retained (Komesidou & Storkel, 2015). During consolidation, which is supported by sleep, recently encoded information is re-activated (Davis & Gaskell, 2009). Words in long-term memory are also activated leading to integration of new and established vocabulary knowledge (James et al., 2017). Through consolidation, activation related to the newly learned words is shifted from the hippocampus to the neocortex and subcortical structures (Davis & Gaskell, 2009). Notably, across gaps in time between periods of input, representations of words evolve. If representations are successfully consolidated, individuals will demonstrate similar knowledge before and after a gap (Leonard & Deevy, 2020). In some cases, individuals perform better on word learning tasks after a gap as newly learned words are integrated with established vocabulary knowledge during sleep (Henderson et al., 2012). Consolidation can be less successful in that individuals demonstrate less knowledge about words after a gap (Malins et al., 2020) or unsuccessful in that word knowledge is forgotten (Storkel, 2015).

If representations of words are consolidated sufficiently, they can be successfully retrieved during the child's next experience with the word. When memories are retrieved, they become malleable and can be re-encoded (McKenzie & Eichenbaum, 2011; Nader & Hardt, 2009). Re-encoding is similar to encoding in that the child builds representations during a period of input. However, during re-encoding the child is refining and adding to a previously encoded representation. It is through the processes of encoding, consolidation, retrieval, and re-encoding across multiple experiences that the child slowly builds robust knowledge of a previously unfamiliar word. This process is referred to as slow- or extended mapping (Swingley, 2010).

Learning a word form to the level that it can be readily retrieved and produced requires extensive experiences with the word, even for children with TD (McGregor et al., 2007). Notably, expressive vocabulary, the ability to retrieve and produce a word form to express meaning, typically lags behind receptive vocabulary, the ability to retrieve a word meaning when given the word form (Bornstein & Hendricks, 2012). However, expressive vocabulary knowledge is essential for effective communication and is linked to both academic and social outcomes throughout childhood (Morgan et al., 2015). Children with TD demonstrate substantial variability in how much experience they need to add a word to their expressive vocabularies (Gray, 2005; McGregor et al., 2007). Currently, it is unclear if this variability is driven by differences in encoding, consolidation, or both. We address this question by assessing children's ability to encode and consolidate word forms during the slow-mapping process.

## Individual Differences in Encoding

Individual differences in working memory abilities affect children's ability to learn words (Archibald, 2017; Gathercole et al., 1997). Verbal working memory includes the ability to encode, temporarily store, and reproduce verbal information (Archibald, 2017). Notably, children's performance in nonword repetition tasks (NWR tasks), in which they must repeat unfamiliar word forms, is related to the rate of vocabulary growth during early childhood (see Gathercole, 2006, for a review). Performance in NWR tasks demonstrates the earliest stage of word form learning, in that the child must temporarily store and reproduce the phonemes that make up the form. Children who encode a more phonologically precise representation after a single exposure have a more phonologically precise representation to build upon during subsequent exposures (Bishop et al., 2012). Thus, they require fewer exposures overall to build a robust representation of the word form.

In addition to verbal working memory abilities, a child's current vocabulary knowledge affects her ability to encode words during periods of input (see Archibald, 2018). When the child encounters an unfamiliar word, related information from long-term memory such as phonologically similar word forms are activated in working memory (Davis & Gaskell, 2009). This activated information supports the temporary storage of the newly encountered information. This is why children more readily encode and repeat novel forms that include sound combinations that are common in their language as opposed to sound combinations that are uncommon (Storkel, 2001). Separating the relative influence of a child's verbal working memory abilities and her current vocabulary knowledge on new word learning is difficult (see Adlof & Patten, 2017) and is not the primary purpose of this study. Instead, we acknowledge that both are likely to influence the encoding of word forms.

## Individual Differences in Consolidation

Currently, little evidence exists on whether children with TD vary in the amount of information they consolidate between periods of input. During consolidation, newly learned words become integrated with known words that are organized in phonological and semantic networks (Gaskell & Dumay, 2003). Thus, children with richer vocabulary knowledge should demonstrate better consolidation than children with poorer vocabulary knowledge. The research team of James, Gaskell, and Henderson have demonstrated that this is the case in some of their studies (James et al., 2017; Henderson & James, 2018), but not in others (James et al., 2020). This limited research on individual differences in consolidation focuses on school-age children. In a notable exception, Henderson et al. (2021) assessed 5- to 7-year-old children's memory for words learned from a storybook when tested immediately and after a period of overnight sleep. Knowledge of the form-referent link was more strongly related to vocabulary knowledge after sleep than before, suggesting better consolidation for children with better vocabulary knowledge. However, no such effect was found for children's ability to produce word forms. Of note, the majority of research on consolidation of word learning includes one training session and one testing session after a delay. However, the slow-mapping process includes interleaving periods of input and consolidation. Currently, it is unclear how consolidation differs across preschool- and kindergarten-age children during the slow-mapping process.

## This Study

The primary goal of this study is to determine whether the rate of word form learning in 4- to 6-year-old children with TD is driven by encoding, consolidation, or both. To address this goal, we trained children who varied in verbal working memory skills and vocabulary knowledge on nine form-referent pairs across subsequent days. Children were trained every day until they demonstrated learning of all words or completed a total of six sessions. As word knowledge gained through instruction is not useful unless it is retained, we assessed children's ability to retrieve word forms after a 1-month posttraining delay. Thus, we assessed if children's ability to retain words over a posttraining delay is an additional source of individual variation in vocabulary growth over time.

We designed each training session to be highly supportive to assess individual differences given a best-case scenario. We provided children with one-on-one instruction via retrieval-based practice because this strategy supports learning and retention for a variety of learners (see Gordon, 2020; Leonard & Deevy, 2020, for reviews). We implemented retrieval-based practice by asking children to retrieve and produce forms throughout training and by providing feedback to their responses (Adesope et al., 2017). We included tasks that varied in retrieval demands in that children were sometimes provided with cues to aid retrieval. This approach is often more effective at supporting learning and retention than only including one type of retrieval task (Adesope et al., 2017). We also utilized spaced practice in that presentations of each form-referent pair were spaced throughout sessions and across sessions. Spaced practice fosters better word learning than massed practice in which presentations of target words are presented close together in time (Fritz et al., 2007).

In Gordon et al. (2021) and this study we implemented a similar methodology. In Gordon et al. (2021), we included children with developmental language disorder (DLD, *n* = 9) and TD (*n* = 9) matched on age, biological sex, and maternal education. Our key question was how children with and without DLD varied in encoding and consolidation during slow mapping. We found that children with DLD encoded less phonologically precise representations of forms than children with TD during periods of input demonstrating weaker encoding abilities. However, children with and without DLD demonstrated a similar ability to retain representations of forms between periods of input demonstrating similar consolidation abilities. They also demonstrated a similar ability to retain forms over a 1-month posttraining delay.

In this study, we included a unique sample of 43 children with TD with a broader range of verbal working memory skills, vocabulary knowledge, and maternal education than Gordon et al. (2021). Our primary question was whether the rate of word learning for children with TD is driven by their ability to encode forms, consolidate forms, or both. As previously noted, verbal working memory skills and vocabulary knowledge are strongly associated with children's word-learning abilities. Thus, our secondary question was how these factors related to children's ability to encode, re-encode, and consolidate forms and to retain forms over the extended posttraining delay. Additionally, as SES is often associated with word-learning abilities (Marulis & Neuman, 2013), we assessed how maternal education was related to word learning during slow mapping.

Through the findings of the current study and Gordon et al. (2021) we will gain an understanding of how variability in memory processes affects word learning in children with TD and DLD. It is important to understand variability across children with TD, in their own right, to inform educational practices. Additionally, understanding TD helps us better understand atypical development and vice versa (Karmiloff-Smith, 1998; Peters & Ansari, 2019). Through gaining a better understanding of the range of abilities of children with TD, we have a better framework for understanding the specificity of word learning challenges for children with DLD. Overall, it is through determining the sources of difficulty for children with poorer word-learning abilities that we can effectively support them through vocabulary instruction and interventions.

## Method

### Participants

All reported protocols and recruitment methods received approval from the institutional review board at Boys Town National Research Hospital. Children were recruited via the Human Subject Research Core participant database. To support database enrollment, speech-language pathologists, and audiologists administered language and hearing screenings during free public events (e.g., library story hour) and home school events, as well as at local child care service providers and kindergarten readiness programs. Social media campaigns, flyer distribution, and word of mouth were also integral tools for recruitment. Parents and/or guardians gave written informed consent for their child to participate. Sessions were conducted in the laboratory of the lead author, at the child's home, at a speech-language clinic operated by Boys Town National Research Hospital, at the child's day care, or in a public place such as a library. Efforts were taken to reduce environmental noise as much as possible during sessions. For example, sessions at public libraries were conducted in a private study room or a quiet section of the library. Families were asked to create a distraction-free environment to the extent possible during sessions that were conducted in participants' homes.

Participants included 43 children between the ages of 4:0–6:11 years (*M*_age_ in months = 66.60, *SD* = 9.36) with 22 girls and 21 boys and with a mean maternal education of 16.63 years (*SD* = 1.89; see Table 1). The reported data was collected between February 2018 and April 2019. All children resided within 30 miles distance of Omaha, Nebraska in the United States during the period of data collection. All children were native speakers of Standard American English with no reported exposure to a second language. Children had no neurological or other developmental disorders, based on parental report. Additionally, all children scored within 1 *SD* for their age on language measures described below. Children's racial/ethnic backgrounds were as follows: White/non-Hispanic = 30, White/Hispanic = 1, Black/non-Hispanic = 2, biracial = 8, and information not provided = 2. The data from one additional child were excluded from analyses due to experimental error.

**Table 1.** Demographic information and standardized test scores.

Variable	Means and standard deviations
Age in months	*M* = 66.60*, SD* = 9.36
	(Min = 51, Max = 83)
Biological sex	*F* = 22, *M* = 21
Maternal education, years of schooling	*M* = 16.63*, SD* = 1.89
	(Min = 12, Max = 20)
WPPSI-IV, Block Design scaled score	*M* = 10.86, *SD* = 2.71
	(Min = 7, Max = 19)
WPPSI-IV, Matrix Reasoning scaled score	*M* = 11.23, *SD* = 2.13
	(Min = 7, Max = 16)
PPVT-4, standardized score	*M* = 114.50, *SD* = 12.82
	(Min = 88, Max = 140)
Nonword repetition, percentage of phonemes correct	*M* = 78.64, *SD* = 10.67
	(Min = 52.08, Max = 92.71)
SPELT-3, standardized score	*M* = 115.51, *SD* = 7.57
	(Min = 98, Max = 130)

*Note.* Means and standard deviations (*SD*) for each score are listed. Minimum and maximum scores are listed in parentheses. Min = minimum; Max = maximum; WPPSI-IV = Wechsler Preschool and Primary Scale of Intelligence–Fourth Edition; PPVT-4 = Peabody Picture Vocabulary Test–Fourth Edition; SPELT-3 = Structured Photographic Expressive Language Test–Third Edition.

### Standardized Tests

Children completed an initial session to determine qualification and to assess language and cognitive abilities. All children demonstrated normal hearing via a pure-tone audiometric screening administered by an audiologist or trained speech-language pathologist. To pass the hearing screening, responses to pure tones presented at 20 dB HL were required in both ears for frequencies of 1–4 kHz. All children demonstrated a typical level of nonverbal IQ by receiving a scaled score of 7 or greater, equivalent to a standardized score of 85, on the Wechsler Preschool and Primary Scale of Intelligence–Fourth Edition (WPPSI-IV; Wechsler, 2012) Block Design and Matrix Reasoning subtests. As productions of forms was a primary outcome variable, children with pronunciation abilities atypical for their age on the Goldman-Fristoe Test of Articulation–Third Edition (GFTA-3; Goldman, 2015) were excluded (children excluded, *n* = 4). For included children, an average of 0.79 (*SD* = 0.94) target forms include phonemes that were not produced correctly on the GFTA-3 (see Supplemental Material S1). However, in many cases these forms resolved in that children produced the forms correctly some time during training. Thus, we elected to code each production of each form based on the phonemes that children actually produced instead of making allowances for items missed on the GFTA-3.

To assess children's language abilities, we administered the following standardized tests: the Peabody Picture Vocabulary Test–Fourth Edition (PPVT-4; Dunn & Dunn, 2007) to assess receptive vocabulary comprehension abilities; the Structured Photographic Expressive Language Test–Third Edition (SPELT-3; Dawson et al., 2003) to capture expressive morphology and syntax skills; and a nonword repetition test (Dollaghan & Campbell, 1998) to capture verbal working memory abilities. The nonword repetition test was coded as the total percentage of phonemes correct. Children were required to achieve a score of 96 or above on the SPELT-3 to participate, as this cutoff demonstrates good sensitivity and specificity for identifying children who do not have DLD (Perona et al., 2005). Standardized measures were administered during the qualification visit with the exception of the PPVT-4 and nonword repetition test that were administered at the end of the 1-month test.

### Stimuli

The stimuli included nine forms created within the laboratory. The form set included three one-syllable forms, /dob/, /mep/, and /plun/; and six two-syllable forms, /bɪnɪp/, /grɑmɚ/, /kinɪt/, /nedɪg/, /sibl̩/, and /topɪn/ (see Appendix A for details about form characteristics). The two-syllable forms contained a consonant–vowel (CV) CV.CVC or CCV.CVC syllable structure. Forms within the set varied in initial consonant or consonant cluster. Each of the target forms had a minimal pair. The minimal pairs for the one-syllable forms varied in the final consonant. For the two-syllable forms, three of the minimal pairs varied from the target in the final consonant, and three varied from the target in the middle consonant. The consonant change in the minimal pair varied in at least two phonetic features from the target consonant. Each form was paired with one of nine objects that were visually distinct from each other in that they varied in size, color, shape, and material (see Appendix A). Throughout training and testing, children never produced the real name for any of the objects, verifying that they were unfamiliar and unnamable to children.

### Assessments of Learning

We used four primary tasks throughout training and delayed testing. These included a free recall production task, a cued recall production task, a four-alternative forced-choice (4AFC) recognition task, and a two-alternative forced-choice (2AFC) recognition task. Note that even though we delegated tasks to training and testing blocks, these tasks both assess children's memory for forms and train children on forms as they were given feedback to their responses throughout blocks. Each session included the free recall task at the beginning of the session to determine what the child had consolidated and retained from the previous session (see Appendix B). This was followed by three training blocks including an explicit labeling block, and two blocks using the 2AFC recognition task. The session ended with two testing blocks to assess what children had encoded during the session. These included a block with the 4AFC task and block with the free and cued recall production tasks. More detail about sessions is included in the protocol section below.

Tasks that varied in cuing were utilized throughout training to support encoding and retention of forms (Adesope et al., 2017). However, free recall tasks in which children are asked to name trained referents most authentically assesses children's representations of forms. In this case children are not given part of the form, as in a cued recall task, or all of the form as in a Recognition task. As free recall tasks often lead to floor performance (Gordon & McGregor, 2014), we administered tasks that varied in cuing at the end of each session to increase sensitivity to assess what children encoded about forms. However, in this study, children's responses to the free recall task proved sensitive to answer our questions. Thus, we used responses to the free recall task administered at the beginning and end of sessions as our outcome variable in all models.

### Free and Cued Recall Tasks

During a trial of the free recall task, the child was shown one of the objects and was asked, “What is this one called?” The child was encouraged to guess if he or she did not produce a verbal response. If the child did not produce the correct form, the child was immediately administered the cued recall probe that included the CV or CCV onset of the target form. For example, if the child was shown the object that was labeled a /plun/ during training and he labeled the object a /nedIg/, he was told, “It starts with /plu/ ….” After the cue was given, children were encouraged to produce the entire form.

Children were provided with feedback contingent to their productions at specific time points in the protocol (see Appendix B). For example, if the child produced the form correctly, the examiner would tell the child, “Yes that's right, this is a /plun/.” If the child provided an incorrect response, the examiner would tell the child, “Actually this a /plun/.” If the child said a phonologically close form (e.g., /plum/ for /plun/) the examiner would respond, “That's really close, but this one is a /plun/.” The child was given a sticker for each correct production to increase motivation and engagement in the task. All children were given a small toy at the end of each session regardless of performance.

### Recognition Tasks

During a given trial of the 2AFC Recognition task, the experimenter placed one of the objects next to a piece of paper that had two large black dots on it (see Appendix C). While pointing to the object, the experimenter asked the child, “What is this one called?” The experimenter then provided the child with two forms to choose from: the target form for the object present, and another form from the set (i.e., distractor form). The experimenter pointed to one of the black dots as he or she produced each form. Children could respond by producing one of the forms, by pointing to one of the dots, or by doing both. The 4AFC dot task was similar, but the child was provided with four options: the target form, the minimal pair of the target, another trained form (distractor form), and a minimal pair of the distractor. Children were provided with feedback in a similar manner to the feedback given during the free and cued recall tasks.

## Protocol

### Training Sessions

Each training session included three testing blocks and three training blocks and lasted approximately 10–15 min (see Appendix B). Throughout a given session, objects were presented in the same order during each block. However, object order was randomized across training sessions. Children completed stretches or walks for 1-min intervals between each block. The first testing block included the free recall task without feedback for all nine form-referent pairs. Any objects that were named correctly were not included in the subsequent training blocks for that session. We implemented this methodology to support children's ability to learn the entire set of form-referent pairs by focusing the training time on the referents they could not name via free recall. Supplemental Material S2 indicates which forms were included in training blocks on each day for each participant. For the majority of forms (87%), once the child produced the form correctly at the beginning of a session, the child produced the form correctly at the beginning of all subsequent sessions.

During Training Block 1, the child was shown each object one at a time and the experimenter labeled the object 2 times (e.g., This is a /bɪnɪp/. You say /bɪnɪp/.). No feedback was provided. During Training Blocks 2 and 3, children completed the 2AFC recognition task with feedback. Training Block 3 was identical to Block 2 with the exception that each target form was paired with a different distractor than Block 2. Children then engaged in two additional Testing Blocks with feedback. During these blocks all form-referent pairs were tested regardless of whether children named them correctly at the beginning of the session. During Testing Block 2, the child completed the 4AFC recognition task with each form-referent pair serving as the target form 1 time and as the alternative form 1 time. To avoid fatigue, we added 1-min breaks in which children stretched or took a short walk between every three trials during this testing block. During the last testing block, children were administered the free and cued recall probes for all objects one at a time. As previously stated, each cued recall probe immediately followed the free recall probe for a specific object if the child did not produce the first CV or CCV of the target form correctly.

Pilot testing revealed that introducing all nine objects one after the other during the first training session was overwhelming for children. Thus, the first training session differed from the other training sessions. The nine form-referent pairs were divided into three sets of three. The first set was introduced via all training and testing blocks included in Appendix B with two exceptions. Testing Block 1 was not administered as children had not yet learned any of the forms. During Training Block 2, each form was presented with a familiar noun (e.g., Is it a /mep/ or a sock?) to familiarize children with the 2AFC Recognition task. After children had completed all blocks with the first set, they completed all blocks with the second and third sets in a similar manner. After all three sets had been presented, children completed Testing Blocks 2 and 3 with all three sets intermixed.

Children continued training sessions, which occurred on subsequent days, until they reached criterion or completed a total of six training sessions. To reach criterion, the child had to demonstrate robust learning of all nine forms and form-referent links at the end of a given session. Thus, for each object, the child either had to respond correctly in the free recall task or had to respond correctly in the cued recall task and had to respond correctly for that same object in the 4AFC recognition task. In Supplemental Material S3, we indicate the total number of training sessions each child completed and the number of words the child achieved criterion with during the last training session.

Across all training days, children were trained on an average of 5.93 (*SD* = 2.32) form-referent pairs per day. An overall average of 5.06 (*SD* = 2.30) forms were cued at the end of each training session after administering the free recall prompt because the child did not produce the first CV or CCV correctly. Session 1 included 12 exposures to each form when the target object was present. For all other sessions, children received nine exposures to forms that were trained and three exposures to forms that were not trained during that session. Across all training days, children received an average of 38.67 (*SD* = 12.52, minimum = 15, maximum = 57) total exposures to each form with the target object present. During each training day, children received up to three additional exposures to forms when the form was included as the alternative form in AFC tasks. See Supplemental Material S4 for more information.

### Long-Term Testing

Children completed testing 1 month after their last training session (range: 25–35 days*, M* = 27.82 days). This session included three testing blocks. During the first block, the experimenter administered a free recall prompt that was immediately followed by a cued recall prompt if the child did not produce the correct initial CV or CCV. No feedback was provided. During the second block, the experimenter administered the 4AFC recognition task for all objects without feedback. The third testing block was identical to the first testing block with the exception that children were given feedback to their responses, similar to the feedback given during training. Children were given 1-min stretch or walk breaks between each block as well as between every three trials of the 4AFC task.

In addition to the 1 month test, children were semirandomly assigned to one of three retest conditions: retest at 1 week posttraining, retest at 2 weeks posttraining, or no retest before the 1 month test. This protocol was implemented to determine if retest at different time intervals improved retrieval at the 1 month test. Semirandom assignment was used to keep the biological sex, ages, and PPVT scores of the three groups relatively balanced. The retest sessions were identical to the 1-month session in that all words were tested in three blocks. However, word order was different than the 1-month session.

## Analyses and Results

### Coding

Each production was independently transcribed by two research assistants into the International Phonetic Alphabet based on high-quality video and audio recordings. Intercoder reliability was 95%. Any disagreements were resolved by viewing the video together. The second author, a certified speech-language pathologist, aided with resolving disagreements when needed. Each production yielded a phonological precision score and a whole word score. For the phonological precision score, the production was compared to the target form to code the number of phonetic features that the child produced correctly. Following the coding system established by Edwards et al. (2004), each consonant was assigned up to three points for the correct productions of place, manner, and voicing features. Each vowel was assigned up to three points for correct productions of backness, height, and tenseness features. As forms varied in the number of phonemes, each production was coded as the total percentage of phonetic features correct. Productions that more closely approximated another form in the set than the target form were coded as zero features correct. For example, the child may state /plum/ for the target form /bɪnɪp/. In this case, /plum/ shares more phonetic features with the trained form, /plun/, than it does with the trained form, /bɪnɪp/. For the whole word score, only productions with 100% of the target phonemes, with no additional phonemes added, were coded as correct. All other productions were coded as incorrect.

### Analyses

All analyses were conducted in an R environment using the lme4 package. For all models, we identified the minimal random effects structure such that random effects that did not significantly improve model fit were omitted. We used Akaike information criterion to determine model fit. For each component of memory, encoding, consolidation, and retention, we fit a model with the log odds of the correct response as the outcome variable using the whole word score and a model with the phonological precision of productions as the outcome variable using the phonological precision score. For the whole word score we used the log odds to account for a lack of homogeneity of variance inherent in analyses with binary responses (see Gordon, 2019). Only responses to the free recall task were included in all models. We contrast coded all dichotomous variables as −.5 and .5 so that model results reflected main effects of all included variables (Brehm & Alday, 2021). All continuous variables were centered in the models.

### Encoding: Whole Word

To determine children's ability to encode word forms from input, we conducted a generalized mixed-effects model with the log odds of a correct response to the free recall task administered at the beginning and end of each training session as the outcome variable. Mixed-effects models include random effects to control for systematic variation in the data when identifying the relationships between the fixed effects and outcome variable (see Gordon, 2019). The random effects of the maximal model included intercepts for participant and word as well as session by participant and session by word slopes. The fixed effects of the maximal model included time (beginning and end of session), training session (2–6), biological sex, and maternal education. Scores from Training Session 1 were excluded as the free recall task was only administered at the end but not the beginning of Session 1. Biological sex was included in all models to increase the interpretation and generalizability of National Institutes of Health–funded research (Arnegard et al., 2020).

Children's responses in the NWR task and children's PPVT (standardized) scores were highly correlated (*r* = .42). Additionally, children's NWR score and age in months were highly correlated (*r* = .44). We could not include all three variables in the same model due to multicollinearity. We elected to include the NWR scores as verbal working memory was likely to be highly related to children's ability to encode words from input. We included a Time × NWR interaction to determine if children with better verbal working memory skills gained more knowledge about words during each session than children with poorer verbal working memory skills. We also fit all models with PPVT standardized scores, and age instead of NWR scores, and the results were very similar (see Supplemental Material S5).

The minimal random effects structure supported by the data included random intercepts for participant and word, and a participant by session slope (see Appendix D, Table D1). Descriptive statistics of the number of words produced correctly at the beginning and end of each training session are listed in Table 2. As training sessions increased the probability of producing a word correctly increased (β = .62, *z* = 11.54, *p* < .0001). On average, children increased the number of words they produced correctly by 1.03 words from one session to the next. There was an effect of time such that the probability of producing words correctly increased from the beginning to the end of the session (β = .73, *z* = 7.98, *p* < .0001). On average, children produced 1.03 more forms correctly at the end of the session in comparison to the beginning of the session. As NWR score increased, the probability of producing the correct form also increased (β = .06, *z* = 3.13, *p* < .01). However, there was not a NWR by time interaction (β = .01, *z* = 1.10, *p* = .27). This indicates that children with a higher NWR score did not increase the probability of a correct response from the beginning to the end of the session more than children with a lower NWR.

**Table 2.** Mean number of words produced with 100% phonological precision (whole word score) and mean phonological precision of productions in response to the free recall task at each assessment point during training.

Number of words produced correctly							
Time	Session 1	Session 2	Session 3	Session 4	Session 5	Session 6	1 Month
Beginning of session		1.35 (1.41)	2.41 (2.10)	3.40 (1.96)	4.15 (2.05)	4.26 (2.66)	4.30 (1.99)
End of session	1.19 (1.28)	2.98 (2.29)	4.10 (2.44)	4.37 (1.91)	4.19 (2.06)	4.53 (2.50)	
**Percentage of phonemic features produced correctly**							
	**Session 1**	**Session 2**	**Session 3**	**Session 4**	**Session 5**	**Session 6**	**1 Month**
Beginning of session		0.22 (0.40)	0.37 (0.46)	0.46 (0.48)	0.53 (0.49)	0.53 (0.49)	0.53 (0.49)
End of session	0.23 (0.40)	0.41 (0.48)	0.53 (0.48)	0.57 (0.48)	0.52 (0.49)	0.57 (0.49)	

*Note.* Standard deviations are included in parentheses.

### Encoding: Phonological Precision

To determine changes in the phonological precision of productions during periods of input, we conducted a linear mixed effects model with the percentage of phonetic features produced correctly in response to the free recall task (see Appendix D, Table D2). The random effects and fixed effects of the maximal model were the same as the previous model. The minimal random effects structure supported by the data included random intercepts for participant and word, and a participant by session slope. The model revealed that as the training sessions increased the percentage of phonetic features produced correctly also increased (β = .10, *t* = 11.96, *p* < .0001). On average, children increased the phonological precision of their productions by 10% from one session to the next. There was an effect of time (β = .12, *t* = 7.87, *p* < .0001) in that children produced forms with 12% more phonological precision at the end of a session in comparison to the beginning of the session. NWR score was positively related to phonological precision of productions (β = .01, *t* = 3.08, *p* < .01). However, there was not a NWR × Time interaction (β = .001, *t* = 0.49, *p* = .63). This indicates that children with varying NWR scores increased the phonological precision of their productions to the same degree from the beginning to the end of a training session.

### Consolidation: Whole Word

To determine children's ability to consolidate forms between periods of input, we conducted a generalized mixed-effects model. The log odds of a correct response to the free recall task at the end and beginning of training sessions was the outcome variable. During training, children had up to five gaps between training sessions (see Figure 1). To assess change across gaps, we coded the productions at the end of Training Session 1 and the beginning of Training Session 2 as Gap 1. We coded all other responses in a similar way. In this case, performance during Gap 1 tells us how children performed after receiving training during session 1, but before receiving training during session 2. Gap 2 tells us how children performed after receiving training during Session 2, but before training during Session 3. The variable Gap is similar to the variable Session from the previous models.^1^

The random effects of the maximal model included intercepts for participant and word as well as Gap by participant and Gap by word slopes. The fixed effects of the maximal model included: Time (end and beginning of session), Gap (1–5), NWR score, biological sex, and maternal education. Performance at the end of each child's last training session was not included in the model as no assessments were conducted the following day. We included a Time × NWR interaction to determine if children with better verbal working memory retained more knowledge about words across periods of consolidation.

The minimal random effects structure supported by the data included random intercepts for participant and word (see Appendix D, Table D3). Participants' performance improved from one Gap to the next (β = .71, *z* = 16.50, *p* < .0001). For example, performance during the second Gap (end of Session 2 and beginning of Session 3) was better than performance during the first Gap (end of Session 1 and beginning of Session 2). There was not an effect of time (β = −.01, *z* = −0.14, *p* = .89) indicating that the probability of producing words correctly did not differ between the end of a session and the beginning of the following session. Participants demonstrated a higher probability of producing a word correctly if they had a higher NWR score (β = .06, *z* = 3.33, *p* < .001). However, there was not a NWR by time interaction (β = −.001, *z* = −.13, *p* = .90) indicating that participants with varying verbal working memory scores did not differ in their ability to consolidate forms during gaps between sessions.

### Consolidation: Phonological Precision

To determine changes in the phonological precision of productions over periods of sleep, we conducted a linear mixed-effects model with the percentage of phonetic features produced correctly in response to the free recall task. The random effects and fixed effects of the maximal model were the same as the previous model. The minimal random effects structure that supported model fit included intercepts for participant and word, gap by participant, and gap by word slopes (see Appendix D, Table D4). Participants increased the percentage of phonetic features produced correctly from one Gap to the next (β = .12, *t* = 14.68, *p* < .0001). There was not an effect of Time (β = .004, *t* = 0.25, *p* = .80) indicating that the phonological precision of productions did not differ between the end of a session and the beginning of the following session. Phonological precision of productions were positively related to NWR score (β = .01, *t* = 2.61, *p* = .01). However, there was not a NWR × Time interaction (β = −.001, *t* = −0.71, *p* = .48) indicating that participants with varying verbal working memory scores did not differ in their ability to consolidate forms.

### Posttraining Retention: Whole Word

We conducted a generalized mixed-effects model to assess the log odds that children would correctly produce forms at the end of their last training session and at the beginning of the 1-month session in the free recall task. The random effects of the maximal model included intercepts for participant and word. The fixed effects of the maximal model included Delay (end of training and beginning of 1-month session), NWR score, biological sex, and maternal education. To assess whether children with better verbal working memory scores retained words better than children with poorer verbal working memory scores, we included a Delay × NWR interaction. We included Retest Condition (1 week, 2 weeks, and no retest) and a Condition × Delay interaction in the model. For Retest Condition, we used Helmert Coding so that model results reflected main effects of all included variables (Schad et al., 2020). Through Helmert Coding, we assessed whether retest at 1 week or 2 weeks led to better retention of learning and whether the two retest conditions led to better retention of learning than the no retest condition.

The minimal random effects structure supported by the data included random intercepts for participant and word (see Appendix D, Table D5). There was an effect of Delay such that children performed worse after the 1-month delay (β = −.66, *z* = −3.91, *p* < .0001). On average, children produced 1.46 fewer words correctly after the 1-month delay. Children with a higher NWR score demonstrated a higher probability of producing words correctly overall (β = .05, *z* = 2.86, *p* < .01). However, there was not an NWR × Delay interaction (β = −.01, *z* = −0.54, *p* = .59). This indicates that children with a higher NWR score did not retain more words than children with a poorer NWR score. There was no Retest Condition × Delay interaction.

### Posttraining Retention: Phonological Precision

To determine changes in the phonological precision of productions across the 1-month delay, we conducted a linear mixed-effects model with the percentage of phonetic features produced correctly in response to the free recall task. The random effects and fixed effects of the maximal model were the same as those listed for the previous model. The minimal random effects structure supported by the data included random intercepts for participant and word (see Appendix D, Table D6). There was an effect of Delay such that children performed worse after the 1-month delay (β = −.13, *t* = −4.39, *p* < .0001). On average, children decreased the phonological precision of their productions by 13%. Children with a higher NWR score produced forms with more phonological precision overall (β = .01, *t* = 2.43, *p* = .02). However, there was not an NWR × Delay interaction (β = −.0001, *t* = −0.02, *p* = .98). There was a Retest Condition × Delay interaction such that children retested at 1 week lost more phonological precision over the 1-month gap than children retested at 2 weeks (β = .09, *t* = 2.36, *p* < .05). However, the phonological precision lost over the 1-month delay did not differ between the no retest condition and the two retest conditions (β = .001, *t* = 0.04, *p* = .98). Descriptive data of children's performance in the retest conditions are listed in Supplemental Material S6.

## Discussion

In this study, we assessed whether individual differences in word learning during slow mapping were driven by differences in encoding, consolidation, or both. We also assessed how the factors verbal working memory, vocabulary knowledge, and maternal education related to word learning during slow mapping. Overall, children demonstrated differences in encoding. Children's verbal working memory skills and vocabulary knowledge were positively related to the number of forms produced and the phonological precision of productions. Notably, children primarily differed in their initially encoded representations of forms. Children's representations of forms improved during periods of input to a similar degree regardless of verbal working memory skills and vocabulary knowledge, demonstrating similar re-encoding abilities. Additionally, children retained information learned about words to a similar degree between periods of input and over an extended posttraining delay. Maternal education was not significantly related to children's ability to encode, consolidate, or retain forms. Key findings are discussed in more detail below.

### Encoding and Re-Encoding

Verbal working memory skills play a critical role in successful word learning in both children and adults (see Gathercole, 2006). One branch of this literature focuses on the association between verbal working memory skills and static vocabulary knowledge (Gathercole et al., 1999). Findings from this research imply, but do not provide direct evidence of, the causal relationship between these two factors. Another branch of this literature focuses on the relationship between verbal working memory skills and dynamic word learning during a single session (Adlof & Patten, 2017; Gathercole et al., 1997). However, this research only captures the earliest stage of word learning. This study provides a bridge between these two bodies of literature by demonstrating how verbal working memory skills relate to children's ability to build stable representations of word forms during the slow-mapping process.

We found that children largely differ in the amount of phonological information they encode during the first learning opportunity. Because of better initial encoding, children with better verbal working memory skills have a more phonologically precise initial representation to build upon. Thus, they are able to achieve phonologically precise representations of forms with fewer overall exposures. These findings are consistent with current literature. Bishop et al. (2012) demonstrated that when individuals repeat unfamiliar forms multiple times during a single session, they differ in the phonological precision of the initial repetition. However, the rate that they refine the phonological precision of the form across subsequent repetitions does not differ across individuals. We observed a similar result in Gordon et al. (2021). Children with DLD had poorer verbal working memory skills than children with TD, and they demonstrated poorer word learning performance overall. However, the rate of growth across training sessions did not differ between groups.

In addition to verbal working memory skills, children's current vocabulary knowledge was related to their ability to encode information about word forms. As previously mentioned, these two factors are highly intertwined throughout development. Children with better verbal working memory abilities more readily add words to their vocabulary from the earliest stages of development (Gathercole, 2006). Additionally, children's vocabulary knowledge supports their ability to encode unfamiliar words (Adlof & Patten, 2017). This highly interactive process is one explanation for the Matthew Effect in which children who know more words can more readily add new words to their vocabularies (Stanovich, 2009). Through further research, we can tease apart the relative influence of these two factors on dynamic word-learning processes. Additionally, we can better understand how other cognitive abilities, such as attention and executive function, interact with verbal working memory and vocabulary knowledge to affect encoding of unfamiliar word forms.

### Consolidation

In this study, individual differences in consolidation did not drive the rate that children learned words during the slow-mapping process. These results are consistent with Gordon et al. (2021) in that children with DLD, who had poorer verbal working memory skills and vocabulary knowledge, did not differ in consolidation abilities from children with TD. As previously mentioned, research investigating individual differences in consolidation of word learning is rare. The work by Henderson et al. is an exception (Henderson et al., 2012, 2013, 2015). They note in Henderson et al. (2015) that the effect of vocabulary knowledge on consolidation is more often revealed in tasks in which words are implicitly taught. Explicit training strategies tend not to demonstrate differences in consolidation across children. Further research is needed to better understand how the nature of the word learning task differentially affects consolidation processes across children. Notably, throughout this body of work, differences in encoding appear to have a larger effect on word learning outcomes than differences in consolidation. Differences in encoding are readily observable in individual studies, but differences in consolidation are inconsistently observed and become more apparent when data are combined across studies (see James et al., 2017).

### Long-Term Retention

In this study, children who varied in working memory skills and vocabulary knowledge did not differ in their ability to retain words over a posttraining delay of 1 month. However, as a group, children produced fewer forms correctly after the 1-month delay. Research on the retention of words across delays of 1 month or longer are rare. Furthermore, these studies tend to include traditional word learning paradigms. Children are trained via one or two sessions and assessments include AFC tasks. Children can retain the form-referent link longer than 1 month under these circumstances (Kan, 2014; Markson & Bloom, 1997). However, the quality of training affects how long word learning is retained. For example, the presence of memory supports such as labeling the object additional times or asking the child to repeat the form increases the probability that the form-referent link will be retained over a 1-month interval (Vlach & Sandhofer, 2012). Assessing the phonological precision of children's representations of forms after extended delays has proven difficult. Kan (2014) assessed productions of forms at the end of training and after 4- and 6-month delays. However, children performed near floor at all time points. To increase sensitivity to measure children's representations, in Gordon et al. (2016), we administered a 3AFC recognition task in which children were given the target form, a minimal pair of the target, and another form. After a single training day, children performed above chance when tested 6 months to 1-year posttraining. However, the length of the delay was negatively related to performance. Thus, even when given an AFC task, children's memory for target forms gradually fades with time.

In this study, we demonstrated that with extended training, children can retrieve and produce forms after a 1-month delay. However, this ability does gradually fade with time. Notably, as we did not train real English words children did not have the opportunity for posttraining exposures through naturalistic interactions. Incidental exposures may be sufficient to help children retain real words after training is withdrawn. However, occasional explicit re-exposures may boost the amount of word knowledge that children retain from training. To this end, we retested words 1 week and 2 weeks after training. Children who were retested 2 weeks after training demonstrated better performance at the 1-month delay than children retested 1 week after training. However, retesting was largely insufficient to boost retention. Children in the two retest conditions performed similarly to children who were not retested. Research with adults has demonstrated that there is a sweet-spot at which words should be retested to lead to optimal performance depending on when the final test is administered (Cepeda et al., 2006). However, educators provide explicit word-learning instruction with the hope that children will retain the word knowledge indefinitely. Further research is needed to identify the optimal spacing of and nature of posttraining tests and reviews that increase the amount of word knowledge children retain long term.

### Educational Implications

The training in this study incorporated many principles of robust vocabulary instruction. Children were taught the same words across multiple sessions via interactive spaced retrieval-based practice. Even given this best-case scenario, children demonstrated marked differences in their ability to encode representations of word forms. It is notable that in 87% of cases, when children produced a form correctly at the beginning of a session, they produced that form correctly at the beginning of all subsequent sessions. This suggests that once children successfully consolidate and can retrieve a form, they are likely to do so at future time points. Understanding the amount and nature of training needed to get children with a variety of language skills to this point is vital. Children are unlikely to improve academic outcomes if they do not consolidate and retain the words they encode during educational instruction.

Current findings confirm past literature in that children with poorer verbal working memory skills and vocabulary knowledge require more exposures than children with better language skills to build robust representations of words (Gray, 2005). It is unclear what the adequate exposures would be to build robust representations in these children, and the dosage is likely to vary across individuals. Confirming this point, Hadley et al. (2021) found an interaction between child and word characteristics in the ability of preschool-age children with TD to learn real words via a classroom-level intervention. Similar to this study, they found an effect of vocabulary knowledge on word-learning ability but did not find an effect of maternal education. We reiterate their conclusion, that more proximal factors such as children's current language skills and quality and quantity of input are more likely to affect children's word-learning outcomes than more distal factors such as maternal education.

The type of one-on-one instruction we provided in this study would be infeasible for classroom-level instruction. However, preschool and kindergarten vocabulary instruction could be improved to provide rich encoding opportunities for all children. Currently, preschool curricula often lack information about how to implement robust vocabulary instruction (Neuman & Dwyer, 2009). This is reflected in real-world practice. Wright and Neuman (2014) observed that in Kindergarten classrooms words are often targeted incidentally instead of being targeted repeatedly and systematically. Classroom-level instruction could be improved by implementing systematic spaced retrieval-based practice thereby increasing the number of explicit exposures to target words (see Beck et al., 2013; Gordon, 2020). This should improve word learning and retention for all children, not only children who demonstrate more difficulty learning words (see Leonard & Deevy, 2020). Furthermore, implementing the tiered approach to instruction is important to prepare all children for formal schooling (Hughes & Dexter, 2011). Specifically, children who are provided with high-quality classroom instruction (Tier 1) but demonstrate slower word learning should be provided with additional support when possible. We can continue to refine Tier 2, small group, and Tier 3, individual level, instruction with the knowledge that encoding word forms is likely to be a key source of difficulty for many children.

## Conclusions

Implementing robust vocabulary instruction in which the same words are targeted across multiple lessons is important to support word learning in 4- to 6-year-old children with a variety of language skills. However, even when given robust instruction individual differences remain. A key difference across children is what they are able to encode from initial instruction. Notably, partnerships between speech-language pathologists and classroom teachers to provide high-quality vocabulary instruction at the classroom level boosts word learning in children with TD and children with language disorders (Throneburg et al., 2000). Through the tiered approach and partnerships between teachers, parents, and speech-language pathologists, children with the poorest word learning skills are more likely to gain and retain vocabulary knowledge during preschool and kindergarten. This approach will optimally prepare children who struggle with word learning for academic and literacy instruction as they enter formal schooling.

## Author Contributions

**Katherine R. Gordon:** Conceptualization (Lead), Data curation (Equal), Formal analysis (Lead), Funding acquisition (Lead), Investigation (Equal), Methodology (Lead), Project administration (Lead), Supervision (Lead), Writing – original draft (Lead), Writing – review & editing (Equal). **Stephanie L. Lowry:** Data curation (Equal), Investigation (Equal), Methodology (Equal), Project administration (Equal), Supervision (Equal), Writing – review & editing (Equal). **Nancy B. Ohlmann:** Resources (Equal), Writing – review & editing (Supporting). **Denis Fitzpatrick:** Software (Lead), Writing – review & editing (Supporting).

## Supplementary Material

10.1044/2022_JSLHR-21-00530SMS1 Supplemental Material S1 Additional information about children's performance on the GFTA-3 and Whole Word and Phonological Precision Scores.

10.1044/2022_JSLHR-21-00530SMS2 Supplemental Material S2 Additional information about words trained each day for each child.

10.1044/2022_JSLHR-21-00530SMS3 Supplemental Material S3 Performance of individual children including the number of words correct at the last training day, and number of words trained to criterion.

10.1044/2022_JSLHR-21-00530SMS4 Supplemental Material S4 Specific characteristics of training days including average number of word trained each day and dosage of words within and across training days.

10.1044/2022_JSLHR-21-00530SMS5 Supplemental Material S5 Final models for all analyses with PPVT scores and final models for all analyses with age in months.

10.1044/2022_JSLHR-21-00530SMS6 Supplemental Material S6 Effect of retest on performance at one month.

## Figures and Tables

**Figure A1.**
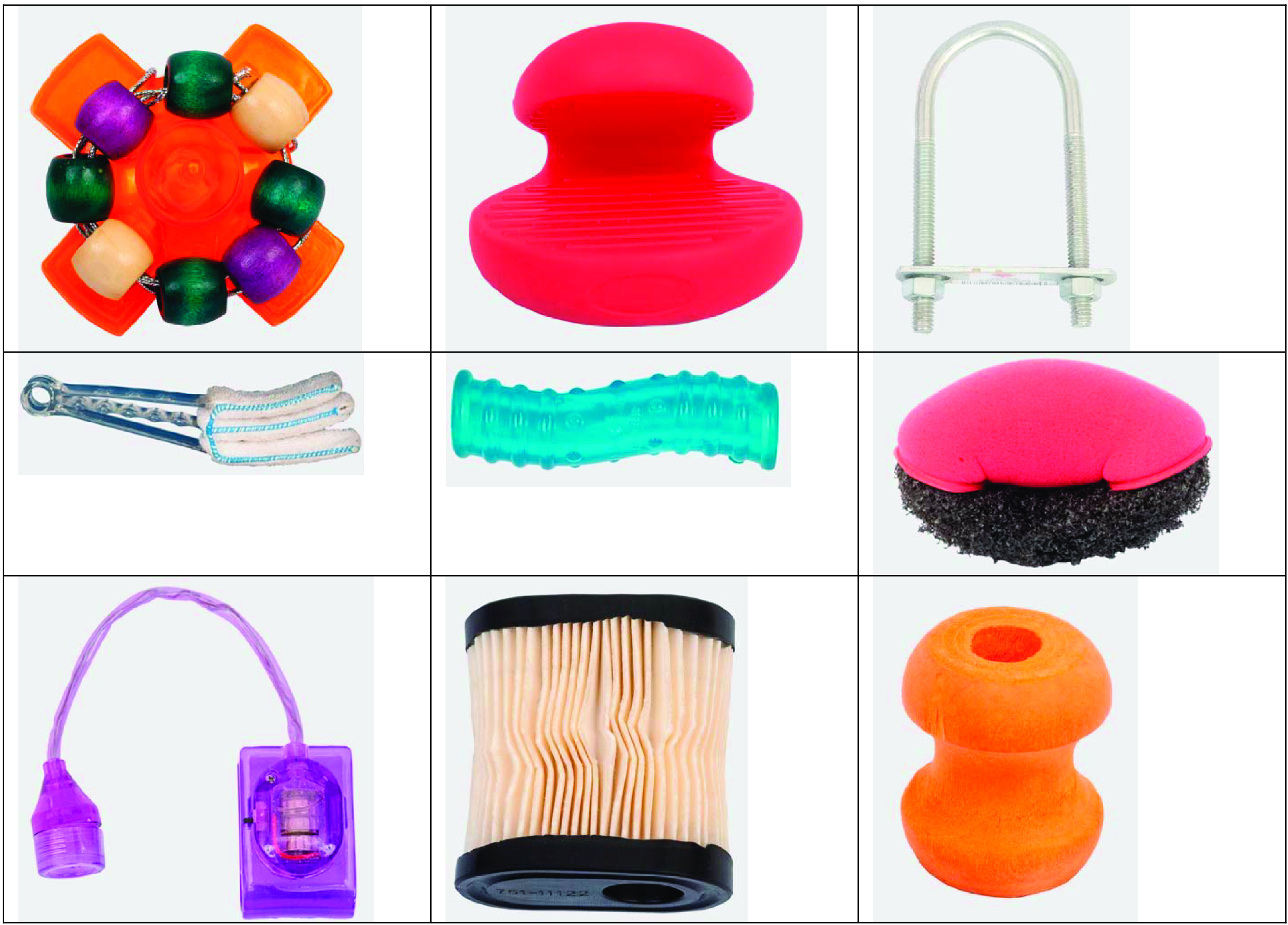
Nine target objects.

**Figure C1.**
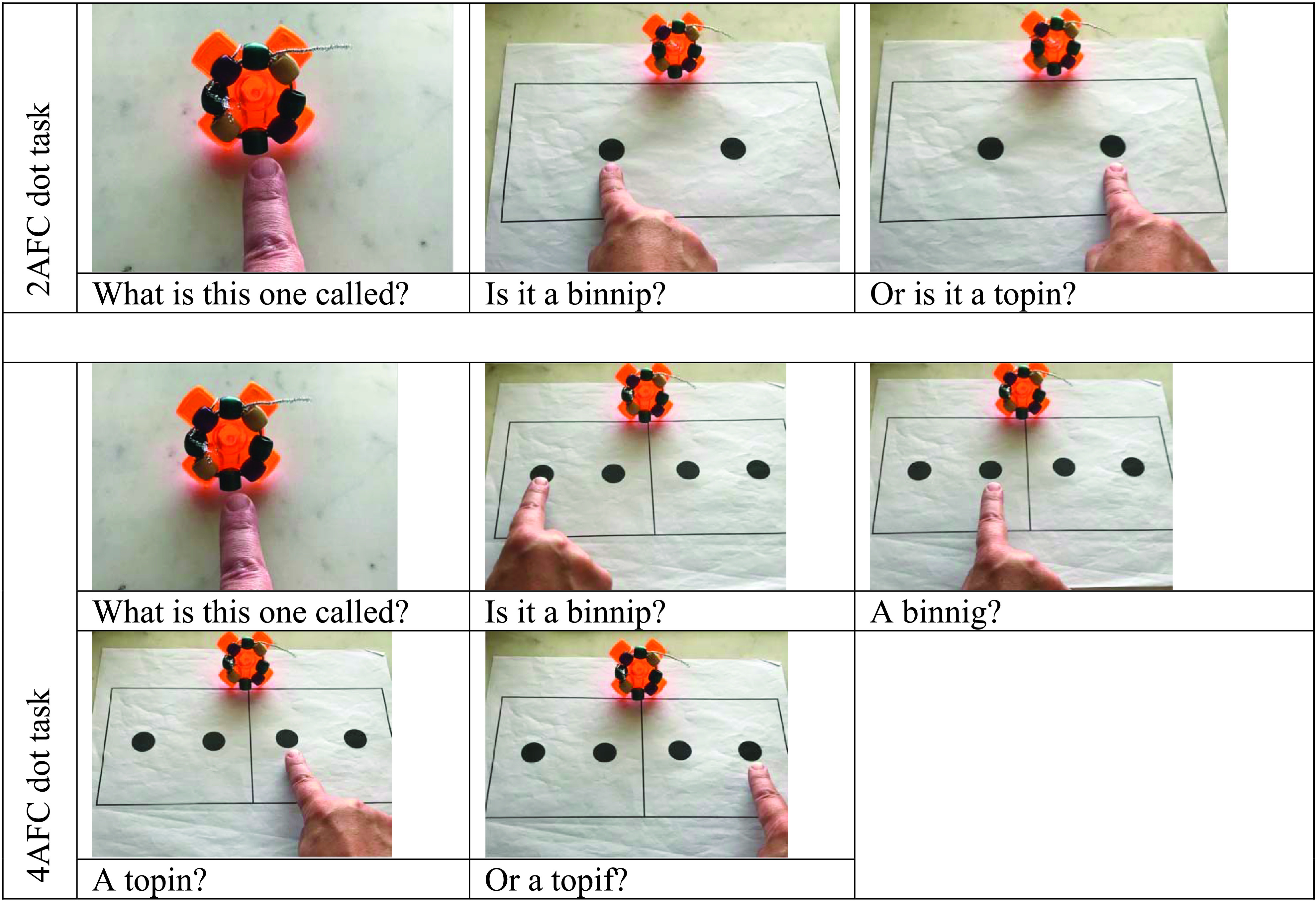
Illustrations of the two-alternative forced-choice (2AFC) and four-alternative forced-choice (4AFC) dot tasks used throughout training and the long-term session.

## Footnote

1 Each session included two assessment points, performance at the beginning and end of the session coded as the variable, Time. In the previous models assessing encoding, the variable Session tells us how children's performance changed from one session to the next. The variable Time tells us how children's performance changed during periods of encoding, from the beginning to the end of sessions. Similarly, each Gap between sessions includes two assessment points, performance at the end of a session and performance at the beginning of the following session coded as the variable Time. In the models assessing consolidation, the variable Gap tells us how children's performance changed from one Gap to the next. The variable, Time, tell us how children's performance changed during periods of consolidation, from the performance at the end a session to performance at the beginning of the next session.

## Appendix A

### Characteristics of Word-Referent Pairs

**Table A1.** Word forms and characteristics, phonotactic probability. Phonotactic probability was calculated with the English calculator (Vitevitch & Luce, 2004; https://calculator.ku.edu/phonotactic/about).

Target form	Phonotactic probability, sum of positional segment frequency	Phonotactic probability, biphone probability	Minimal pair	Phonotactic probability, sum of positional segment frequency	Phonotactic probability, biphone probability
/bɪnɪp/	.3117	.0191	/bɪnɪg/	.303	.0197
/dob/	.1271	.0027	/dof/	.1208	.0016
/grɑmɚ/	.2104	.0186	/grɑtɚ/	.2703	.0250
/kinɪt/	.3488	.0093	/kigɪt/	.2706	.0033
/mep/	.1235	.0045	/mev/	.11	.0043
/nedɪg/	.1505	.0099	/nedɪp/	.1592	.0093
/plun/	.1905	.0092	/plub/	.1617	.0088
/sibl̩/	.183	.0061	/sifl̩/	.1767	.0041
/topɪn/	.2547	.0078	/topɪf/	.1929	.0058

**Table A2.** Word forms and characteristics, neighborhood density. Neighborhood density was calculated using the English child corpus calculator (Vitevitch & Luce, 2004; https://calculator.ku.edu/density/about).

Target form	Neighborhood density	Minimal pair	Neighborhood density
/bɪnɪp/	0	/bɪnɪg/	0
/dob/	7	/dof/	7
/grɑmɚ/	0	/grɑtɚ/	0
/kinɪt/	0	/kigɪt/	0
/mep/	13	/mev/	15
/nedɪg/	0	/nedɪp/	0
/plun/	2	/plub/	0
/sibl̩/	2	/sifl̩/	0
/topɪn/	0	/topɪf/	0

## Appendix B

### Training and Testing Blocks

**Table B1.** Protocol during each training session.

Block	Task	Objects	Script	Feedback
Testing Block 1	Free recall	All objects	What is this one called?	None
Training Block 1	Explicit labeling	Only objects that were not named correctly during testing Block 1	This is a /bɪnɪp/. You say /bɪnɪp/.	None
Training Block 2	2AFC dot task	Only objects that were not named correctly during testing Block 1	Is this one a /bɪnɪp/ or a /mep/?	Correct = “Yes that's right this is a /bɪnɪp/.” Incorrect = “Actually this is called a /bɪnɪp/.” Incorrect/close = “That's really close, but this one is a /bɪnɪp/.”
Training Block 3	2AFC dot task	Only objects that were not named correctly during testing Block 1	Is this one a /gramɚ/ or a /bɪnɪp/?	The same as training Block 2
Testing Block 2	4AFC dot task	All objects	Is this one a /bɪnɪp/, a /bɪnɪg/, a /dob/, or a /dof/?	The same as training Block 2
Testing Block 3	Free/cued recall test	All objects	What is this one called? It starts with /bɪ/…	The same as training Block 2

*Note.* 2AFC = two-alternative forced-choice; 4AFC = four-alternative forced-choice.

## Appendix C

### The 2AFC and 4AFC Dot Tasks

**Description of Tasks**

In a given trial of the 2AFC dot task, an object as well as a piece of paper with two large dots on it were placed in front of the child. The experimenter first pointed to the object and asked, “What is this one called?” The experimenter then presented the child with two forms to choose from, the target form for the object present and another trained form from the set. As she produced the first form the experimenter pointed to one of the black dots on the paper. As she produced the second form she pointed to the other black dot. Children could indicate their response by producing one of the forms, by pointing to one of the black dots, or by doing both. If the child produced a form and pointed to the dot not associated with that form, their stated form took precedence. If the child said a form that was phonologically similar but not identical to one of the options, this was coded as a production of the target form (e.g., /meb/ for /mep/). If the child produced a form that was not phonologically similar to either of the options or blended two forms, the experimenter re-administered the trial and encouraged the child to select one of the stated options. The pairing of target and distractor forms as well as the order they were presented within trials (e.g., target form produced first or second) were randomized throughout training. Feedback of the correct form after each trial was given in a manner similar to the feedback provided during the free and cued recall tasks. For example, if the child produced the form correctly, the examiner would tell the child, “Yes that's right, this is a /plun/.” If the child provided an incorrect response, the examiner would tell the child, “Actually this a /plun/.” If the child said a phonologically close form (e.g., /plum/ for /plun/) the examiner would respond, “That's really close, but this one is a /plun/.”

The four-alternative forced-choice (4AFC) dot task was similar to the two-alternative forced-choice (2AFC) dot task, but each trial included four forms as options: the target form, a minimal pair of the target, another trained form (i.e., the distractor form), and a minimal pair of the distractor form. The 4AFC dot task was administered with the object placed at the top of a paper that included four large dots on it arranged with two dots each in two large squares. For example, the experimenter would show the child the object labeled as a /mep/ during training, and would ask the child, “What is this one called? Is it a /mep/, a /mev/, a /bɪnɪg/, or a /bɪnɪp/?” The experimenter pointed to each dot, left to right from the child's point of view, simultaneous to the production of each form. The order that the four options were presented within trials was balanced such that the correct response was presented a similar number of times in each position (produced first, second, third, or fourth). The minimal pair for each form was always either produced directly before or directly after that form. Feedback mirrored what was given for the 2AFC dot task. Responses were coded in the same way as the 2AFC dot task.

## Appendix D

### Final Models for All Analyses With Nonword Repetition (NWR) Scores

**Table D1.** Final model for word learning performance across training days, whole word score.

Log odds of producing a word correctly				
Variable	Estimate	Standard error	*z* value	Pr(>|*z*|)
Intercept	−1.42	0.32	−4.40	< 0.0001
Session^a^	0.62	0.05	11.54	< 0.0001
Time^b^	0.73	0.09	7.98	< 0.0001
NWR	0.06	0.02	3.13	< 0.01
NWR × Time	0.01	0.01	1.10	0.27
Biological sex^c^	0.45	0.36	1.25	0.21
Maternal education	0.09	0.09	0.93	0.35

a Session 2 was coded as 0 to reflect performance at the intercept.

b Beginning of session was coded as −.5. End of session was coded as .5.

c Female was coded as −.5. Male was coded as .5.

**Table D2.** Final model for word learning performance across training days, phonological precision score.

Percentage of phonetic features produced correctly				
Variable	Estimate	Standard error	*t* value	Pr(>|*z*|)
Intercept	0.34	0.06	5.73	< 0.0001
Session^a^	0.10	0.01	11.96	< 0.0001
Time^b^	0.12	0.01	7.87	< 0.0001
NWR	0.01	0.003	3.08	< 0.01
NWR × Time	0.001	0.001	0.49	0.63
Biological sex^c^	0.10	0.06	1.64	0.11
Maternal education	0.02	0.02	1.55	0.13

a Session 2 was coded as 0 to reflect performance at the intercept.

b Beginning of session was coded as −.5. End of session was coded as .5.

c Female was coded as −.5. Male was coded as .5.

**Table D3.** Final model for consolidation during training, whole word score.

Log odds of producing a word correctly				
Variable	Estimate	Standard error	*z* value	Pr(>|*z*|)
Intercept	−2.04	0.33	−6.23	< 0.0001
Gap^a^	0.71	0.04	16.50	< 0.0001
Time^b^	−0.01	0.09	−0.14	0.89
NWR	0.06	0.02	3.33	< 0.001
NWR × Time	−0.001	0.01	−0.13	0.90
Biological sex^c^	0.40	0.35	1.17	0.24
Maternal education	0.08	0.09	0.90	0.37

a Gap 1 was coded as 0 to reflect performance at the intercept.

b Beginning of session was coded as −.5. End of session was coded as .5.

c Female was coded as −.5. Male was coded as .5.

**Table D4.** Final model for consolidation during training, phonological precision score.

Percentage of phonetic features produced correctly				
Variable	Estimate	Standard error	*t* value	Pr(>|*z*|)
Intercept	0.26	0.06	4.57	< 0.0010
Gap^a^	0.12	0.01	14.68	< 0.0001
Time^b^	0.004	0.02	0.25	0.80
NWR	0.01	0.002	2.61	0.01
NWR × Time	−0.001	0.001	−0.71	0.48
Biological sex^c^	0.07	0.05	1.40	0.17
Maternal education	0.02	0.01	1.51	0.14

a Gap 1 was coded as 0 to reflect performance at the intercept.

b Beginning of session was coded as −.5. End of session was coded as .5.

c Female was coded as −.5. Male was coded as .5.

**Table D5.** Final model assessing the effect of delay (end of training and 1-month delay) on production of words, whole word score.

Log odds of producing a word correctly				
Variable	Estimate	Standard error	*z* value	Pr(>|*z*|)
Intercept	0.19	0.31	0.61	0.54
Delay^a^	−0.66	0.17	−3.91	< 0.0001
NWR	0.05	0.02	2.86	< 0.01
NWR × Delay	−0.01	0.02	−0.54	0.59
Biological sex^b^	0.26	0.33	0.78	0.44
Condition, contrast 1^c^	0.01	0.20	0.06	0.95
Condition, contrast 2^d^	0.14	0.11	1.25	0.21
Condition × Delay, contrast 1	0.33	0.21	1.58	0.11
Condition × Delay, contrast 2	−0.06	0.12	−0.50	0.62
Maternal education	0.13	0.09	1.55	0.12

a End of last training session was coded as −.5. Beginning of 1-month session was coded as .5.

b Female was coded as −.5. Male was coded as .5.

c Retest at 1 week was coded as −1, Retest at 2 weeks was coded as 1, and No retest was coded as 0.

d Retest at 1 week was coded as −1, Retest at 2 weeks was coded as −1, and No retest was coded as 2.

**Table D6.** Final model assessing the effect of delay (end of training and 1-month delay) on production of words, phonological precision score.

Percentage of phonetic features produced correctly				
	Estimate	Standard error	*t* value	Pr(>|*z*|)
Intercept	0.59	0.06	9.52	< 0.0001
Delay^a^	−0.13	0.03	−4.39	< 0.0001
NWR	0.01	0.003	2.43	0.02
NWR × Delay	−0.0001	0.003	−0.02	0.98
Biological sex^b^	0.08	0.07	1.17	0.25
Condition, contrast 1^c^	−0.01	0.04	−0.19	0.85
Condition, contrast 2^d^	0.02	0.02	1.09	0.28
Condition × Delay, contrast 1	0.09	0.04	2.36	0.02
Condition × Delay, contrast 2	0.001	0.02	0.04	0.97
Maternal education	0.03	0.02	1.64	0.11

a End of last training session was coded as −.5. Beginning of 1-month session was coded as .5.

b Female was coded as −.5. Male was coded as .5.

c Retest at 1 week was coded as −1, Retest at 2 weeks was coded as 1, and No retest was coded as 0.

d Retest at 1 week was coded as −1, Retest at 2 weeks was coded as −1, and No retest was coded as 2.
