# Supplementary material for: Word Learning by Preschool-Age Children: Differences in Encoding, Re-Encoding, and Consolidation Across Learners During Slow Mapping
Source: J Speech Lang Hear Res. 2022 Apr 20;65(5):1956–77. doi: 10.1044/2022_JSLHR-21-00530 (PMC9559666; doi:10.1044/2022_JSLHR-21-00530)
Supplement: Supplemental Material S1 [file JSLHR-65-1956-s001.pdf]

Supplemental Material S1. Additional information about children's performance on the GFTA-3 and Whole Word and Phonological Precision Scores.

Below we list each item on the Goldman-Fristoe Test of Articulation – 3 (GFTA-3) that are relevant to our target word forms (Table 1). In column 1, we list all target forms, and in columns 2-3 we list GFTA-3 items related to each target form.

Table S1.1. GFTA-3 items that are related to target forms included in the current study.

**Target Form**	**GFTA Correspondence**		
	**Initial Word Position**	**Medial Word Position**	**Final Word Position**
/mep/	11: monkey		4: cup<br>26: soap
/plun/	18: plate		24: lion<br>45: green<br>53: crown<br>60: seven
/dob/	2: door<br>8: duck		16: web
/grɑmɚ/	45: green	12: hammer<br>50: pajamas	12: hammer<br>15: spider<br>28: tiger<br>30: finger<br>36: teacher<br>43: brother
/kinɪt/	4: cup<br>48: cookie	52: princess	18: plate<br>46: that
/nedɪg/	19: knife	15: spider	3: pig<br>44: frog
/topɪn/	10: table<br>28: tiger<br>36: teacher<br>51: teeth	6: apple	24: lion<br>45: green<br>53: crown<br>60: seven
/bɪnɪp/	5: boy	52: princess	4: cup<br>26: soap
/sibḷ/	26: soap<br>60: seven		10: table<br>39: vegetable

In Table 2 we list GFTA-3 items that children missed that are related to target forms. Column 2 includes the item from the GFTA-3 that the child produced in error. The underlined phoneme of the GFTA-3 item is the relevant phoneme to the target word form for initial, medial, or final position of each word form. The child production of the underlined portion is listed in parenthesis afterwards. Column 3 lists the target word form related to the GFTA-3 items. Column 4 indicates the child's best production of the target form at any time during training or long-term recall sessions. This is followed by the percentage of phonetic features correct.

Table S1.2. Items missed on the GFTA-3 that were related to phonemes in the target words.

Participant	Goldman-Fristoe Items	Target Word Form	Best Production
16C0368	45: green (/gw/)<br>12: hammer (child omission)<br>15: spider (/ə/)<br>30: finger child omission)<br>36: teacher (child omission)<br>43: brother (child omission)	/grɑmɚ/	/grɑmʊ/ = 72%
16C0377	60: seven (/θ/)	/sibl̩ /	/sibl̩ / = 100%
16C0409	18: plate (/p-/)	/plun /	/pwun / = 92%
	28: tiger (/ə/)<br>30: finger (/ə/)<br>43: brother (/ə/)	/grɑmɚ/	/grɑbɚ/ = 94%
17C0426	26: soap (/θ/)<br>60: seven (/θ/)	/sibl̩/	/sibl̩/ = 100%
18J0475	10: table (/bo/)<br>39: vegetable (/bo/)	/sibl̩/	/sibl̩/ = 100%
18J0478	45: green (/gw/)<br>12: hammer (/ə/)<br>15: spider (/ə/)	/grɑmɚ/	/gwɑmʊ/ = 67%
	26: soap (/θ/)	/sibl̩/	/sibl̩/ = 100%
18J0487	18: plate (/pw/)	/plun/	/plun/ = 100%
	45: green (/gw/)	/grɑmɚ/	/gwɑmʊ/ = 67%
	39: vegetable (/bo/)	/sibl̩/	/sibl̩/ = 100%
18J0488	36: teacher (/ə/)	/grɑmɚ/	/gwɑmʊ/ = 67%
	39: vegetable (/bo/)	/sibl̩/	/sibl̩/ = 100%
18J0518	45: green (/gw/)<br>12: hammer (/ʌ/)<br>15: spider (/ʌ/)<br>28: tiger (/o/)<br>30: finger (/ʌ/)<br>36: teacher (/ʌ /)<br>43: brother (/ʌ /)	/grɑmɚ/	/gwɑmʊ/ = 67%
	39: vegetable (/vl/)	/sibl̩/	/sibl̩/ = 100%
18J0519	45: green (/gw/)<br>12: hammer (child omission)<br>15: spider (child omission)	/grɑmɚ/	/gwɑmʊ/ = 67%

	28: tiger (child omission)<br>30: finger (child omission)<br>36: teacher (child omission)<br>43: brother (child omission)		
18J0539	18: plate (/pw/)	/plun/	/plun/ = 100%
	26: soap (/θ/)<br>60: seven (/θ/)	/sibl̩/	/sibl̩/ = 100%
18J0540	10: table (/bo/)	/sibl̩/	/sibl̩/ = 100%
18J0551	18: plate (/pw/)	/plun/	/plum/ = 92%
	45: green (/gw/)<br>12: hammer (/ə/)<br>15: spider (/ə/)<br>28: tiger (/ə/)<br>30: finger (/ə/)<br>36: teacher (/ə/)<br>43: brother (/ə/)	/grɑmɚ/	/gwɑmʊ/ = 67%
18J0567	18: plate (/pw/)	/plun/	/plun/ = 100%
	45: green (/gw/)	/grɑmɚ/	/gwɑmɚ/ = 94%
18J0573	45: green (/gw/)<br>12: hammer (/o/)<br>15: spider (/ə/)<br>28: tiger (/ə/)<br>30: finger (/ə/)<br>43: brother (/ə/)	/grɑmɚ/	/grɑmʊ/ = 72%
19J0589	18: plate (/fl/)	/plun/	/plun/ = 100%
19J0593	12: hammer (/o/)<br>15: spider (/ə/)<br>28: tiger (/ə/)<br>30: finger (/o/)<br>36: teacher (/o/)<br>43: brother (/o/)	/grɑmɚ/	/gwɑmʊ/ = 67%
19J0598	18: plate (/pj/)	/plun/	/plun/ = 100%
	45: green (/gw/)<br>12: hammer (/ə/)<br>15: spider (/ə/)<br>28: tiger (/ə/)<br>30: finger (/ə/)<br>36: teacher (/ə/)<br>43: brother (/o/)	/grɑmɚ/	/gwɑmʊ/ = 67%
19J0602	18: plate (/pw/)	/plun/	/plun/ = 100%
	45: green (/gw/)<br>12: hammer (/ə/)	/grɑmɚ/	/gwɑmʊ/ =67%

	15: spider (/ə/)<br>28: tiger (/ə/)<br>30: finger (/o/)<br>36: teacher (/o/)<br>43: brother (/ə/)		
	10: table (/bo/)	/sibl̩/	/θibl̩/ = 100%
19J0604	45: green (/gw/)	/grɑmɚ/	/gwɑmɚ/ = 94%
	10: table (/bo/)	/sibl̩/	/sibl̩/ = 100%
19J0605	26: soap (/θ/)<br>60: seven (/θ/)	/sibl̩/	/sibl̩/ = 100%

Average best production and standard deviation in paratheses	Mean = .87 (.15)

*No errors on items on GFTA-3 that were related to training forms: 17C0406, 17J0453, 17J0455, 18J0479, 18J0482, 18J0522, 18J0538, 18J0541, 18J0542, 18J0555, 18J0556, 18J0560, 18J0565, 18J0568, 18J0571, 18J0572, 18J0574, 19J0588, 19J0595, 19J0597, 19J0599, 19J0601.

Additional Information about Whole Word and Phonological Precision Scores Coding

One form, /grɑmɚ/, proved to be considerably difficult to articulate for multiple children. The phonological process of liquid simplification in the initial consonant cluster and final /ɚ/, or any combination there-of, was accepted as a correct verbal response. This resulted in forms such as /gwɑmʊ/ə/, /grɑmʊ/ə/, and /gwɑmɚ/ being reinforced with affirmative verbal examiner feedback. However, the examiner would produce the correct target articulation of the form (i.e., "Yes, that's right. This is a /grɑmɚ/"). If the child omitted /r/ in the initial consonant cluster or final /ɚ/, these forms were counted as errors. If this occurred during the beginning of session free recall task, children were trained on this form. If this occurred at the end of session free recall task, children received the initial CV cue. However, these variations were counted as correct when determining criterion, in that children retrieved and produced all forms correctly at the end of one training day. Some children were noted to resolve these articulation errors as they demonstrated increased learning of the form. However, not all children demonstrated articulation resolve by the time they reached criteria for all other forms, or by the end of the sixth training day. For coding, children's productions of this form were coded in the same way as all other forms.

For the coding of all forms, when children produced the phonetic features of the target form with 100% accuracy but produced additional phonemes, this production was coded as incorrect for the whole word score. This was a strict coding of whether children fully knew the form in that they could successfully retrieve it and produce it with complete accuracy. However, this production was coded as 100% features produced correctly for the phonological precision score because they could successfully retrieve and produce all phonemes of the target form. This type of error was incredibly rare. It only occurred for twenty-five productions across all children at all time points.
