# Supplementary material for: Word Learning by Preschool-Age Children: Differences in Encoding, Re-Encoding, and Consolidation Across Learners During Slow Mapping
Source: J Speech Lang Hear Res. 2022 Apr 20;65(5):1956–77. doi: 10.1044/2022_JSLHR-21-00530 (PMC9559666; doi:10.1044/2022_JSLHR-21-00530)
Supplement: Supplemental Material S2 [file JSLHR-65-1956-s002.pdf]

Supplemental Material S2. Additional information about words trained each day for each child.

Table S2.1. Words that were retrieved and produced correctly in response to the Free Recall task at the beginning of each session. These words did not receive training during Training Blocks 1–3 on the day indicated. All words were trained for all children on Day 1.

Participant	Day 2	Day 3	Day 4	Day 5	Day 6
16C0368	/sibl̩/	/sibl̩/	/dob/<br>/grɑmɚ/<br>/sibl̩/	/dob/<br>/grɑmɚ/<br>/kinɪt/<br>/mep/<br>/nedɪg/<br>/plun/<br>/sibl̩/	
16C0377	/grɑmɚ/	/dob/<br>/grɑmɚ/<br>/mep/	/dob/<br>/grɑmɚ/<br>/mep/<br>/nedɪg/<br>/plun/<br>/sibl̩/		
16C0409		/mep/<br>/sibl̩/	/grɑmɚ/<br>/sibl̩/	/dob/<br>/grɑmɚ/<br>/mep/<br>/plun/<br>/sibl̩/<br>/topɪn/	
17C0406		/sibl̩/	/dob/<br>/grɑmɚ/<br>/mep/<br>/sibl̩/	/grɑmɚ/<br>/mep/<br>/sibl̩/	/dob/<br>/mep/<br>/plun/<br>/sibl̩/
17C0426		/sibl̩/	/kinɪt/<br>/mep/	/bɪnɪp/<br>/dob/<br>/kinɪt/<br>/mep/<br>/sibl̩/	
17J0453		/dob/<br>/grɑmɚ/<br>/plun/<br>/sibl̩/	/dob/<br>/grɑmɚ/<br>/kinɪt/<br>/mep/<br>/plun/<br>/sibl̩/	/dob/<br>/grɑmɚ/<br>/kinɪt/<br>/mep/<br>/plun/<br>/sibl̩/	/bɪnɪp/<br>/dob/<br>/grɑmɚ/<br>/kinɪt/<br>/mep/<br>/nedɪg/<br>/plun/<br>/sibl̩/
17J0455	/dob/	/dob/	/dob/<br>/nedɪg/<br>/plun/<br>/sibl̩/	/dob/<br>/grɑmɚ/<br>/mep/<br>/nedɪg/<br>/plun/<br>/sibl̩/	/dob/<br>/grɑmɚ/<br>/kinɪt/<br>/nedɪg/<br>/plun/<br>/sibl̩/
18J0475	/dob/<br>/grɑmɚ/	/dob/<br>/grɑmɚ/	/dob/<br>/grɑmɚ/<br>/kinɪt/<br>/sibl̩/	/bɪnɪp/<br>/dob/<br>/grɑmɚ/<br>/kinɪt/<br>/plun/	

				/sibl̩/<br>/topɪn/	
*18J0478	/sibl̩/	/dob/<br>/sibl̩/	/dob/<br>/kinɪt/<br>/mep/<br>/sibl̩/	/dob/<br>/grɑmɚ/<br>/kinɪt/<br>/sibl̩/	/dob/<br>/grɑmɚ/<br>/kinɪt/<br>/sibl̩/
18J0479	/plun/	/mep/<br>/nedɪg/<br>/plun/	/dob/<br>/mep/<br>/nedɪg/<br>/plun/<br>/sibl̩/		
18J0482	/dob/<br>/grɑmɚ/<br>/kinɪt/<br>/plun/<br>/sibl̩/				
18J0487		/plun/	/plun/	/bɪnɪp/<br>/grɑmɚ/<br>/kinɪt/<br>/plun/<br>/sibl̩/	
18J0488	/sibl̩/	/bɪnɪp/<br>/dob/<br>/grɑmɚ/<br>/kinɪt/<br>/plun/<br>/sibl̩/<br>/topɪn/			
**18J0518	/dob/<br>/kinɪt/<br>/nedɪg/<br>/topɪn/	/grɑmɚ/<br>/kinɪt/<br>/nedɪg/<br>/sibl̩/<br>/topɪn/	/bɪnɪp/<br>/grɑmɚ/<br>/kinɪt/<br>/mep/<br>/nedɪg/<br>/plun/<br>/sibl̩/<br>/topɪn/	/bɪnɪp/<br>/dob/<br>/grɑmɚ/<br>/kinɪt/<br>/mep/<br>/nedɪg/<br>/plun/<br>/sibl̩/<br>/topɪn/	
18J0519			/dob/<br>/grɑmɚ/<br>/plun/<br>/sibl̩/	/dob/<br>/grɑmɚ/<br>/kinɪt/<br>/sibl̩/	/dob/<br>/grɑmɚ/<br>/plun/<br>/sibl̩/
18J0522	/dob/<br>/grɑmɚ/<br>/sibl̩/	/dob/<br>/grɑmɚ/<br>/kinɪt/<br>/mep/<br>/nedɪg/<br>/sibl̩/			
18J0538	/grɑmɚ/<br>/kinɪt/<br>/nedɪg/	/grɑmɚ/<br>/kinɪt/<br>/nedɪg/<br>/sibl̩/	/dob/<br>/grɑmɚ/<br>/kinɪt/<br>/nedɪg/<br>/plun/	/dob/<br>/grɑmɚ/<br>/kinɪt/<br>/nedɪg/<br>/sibl̩/	/dob/<br>/grɑmɚ/<br>/kinɪt/<br>/nedɪg/<br>/plun/

			/sibl̩/		/sibl̩/<br>/topɪn/
18J0539	/dob/<br>/gramɚ/<br>/topɪn/	/dob/<br>/gramɚ/<br>/nedɪg/<br>/plun/<br>/sibl̩/<br>/topɪn/	/dob/<br>/gramɚ/<br>/mep/<br>/nedɪg/<br>/plun/<br>/sibl̩/<br>/topɪn/		
18J0540				/mep/	
18J0541	/dob/<br>/gramɚ/<br>/nedɪg/<br>/plun/<br>/sibl̩/	/bɪnɪp/<br>/dob/<br>/gramɚ/<br>/kinɪt/<br>/mep/<br>/nedɪg/<br>/plun/<br>/sibl̩/			
***18J0542	/dob/<br>/sibl̩/	/dob/<br>/gramɚ/<br>/nedɪg/<br>/sibl̩/	/gramɚ/<br>/kinɪt/<br>/nedɪg/<br>/plun/<br>/sibl̩/	/gramɚ/<br>/kinɪt/<br>/nedɪg/<br>/plun/<br>/sibl̩/	/bɪnɪp/<br>/dob/<br>/gramɚ/<br>/kinɪt/<br>/nedɪg/<br>/plun/<br>/sibl̩/
18J0551			/kinɪt/	/gramɚ/<br>/kinɪt/<br>/topɪn/	/gramɚ/<br>/kinɪt/
18J0555	/dob/<br>/kinɪt/	/dob/<br>/gramɚ/<br>/kinɪt/<br>/mep/<br>/plun/<br>/sibl̩/			
18J0556	/dob/<br>/plun/<br>/sibl̩/				
18J0560	/dob/	/dob/	/dob/<br>/gramɚ/<br>/nedɪg/	/dob/<br>/gramɚ/<br>/mep/<br>/nedɪg/	/dob/<br>/gramɚ/
18J0565			/plun/	/gramɚ/<br>/mep/<br>/plun/	/gramɚ/<br>/mep/<br>/plun/
18J0567	/dob/	/dob/<br>/gramɚ/<br>/sibl̩/	/bɪnɪp/<br>/dob/<br>/gramɚ/<br>/kinɪt/<br>/mep/<br>/nedɪg/<br>/sibl̩/		
18J0568	/dob/<br>/plun/<br>/topɪn/	/bɪnɪp/<br>/dob/<br>/kinɪt/<br>/mep/	/bɪnɪp/<br>/dob/<br>/gramɚ/<br>/mep/	/bɪnɪp/<br>/dob/<br>/gramɚ/<br>/kinɪt/	/bɪnɪp/<br>/dob/<br>/gramɚ/<br>/kinɪt/

		/plun/<br>/topɪn/	/sibl̩/<br>/topɪn/	/mep/<br>/nedɪg/<br>/plun/<br>/sibl̩/<br>/topɪn/	/mep/<br>/nedɪg/<br>/plun/<br>/sibl̩/<br>/topɪn/
18J0571		/nedɪg/<br>/plun/	/nedɪg/<br>/plun/	/mep/<br>/nedɪg/<br>/plun/<br>/sibl̩/	/bɪnɪp/<br>/dob/<br>/kinɪt/<br>/nedɪg/<br>/plun/<br>/sibl̩/
18J0572	/kinɪt/	/dob/	/dob/<br>/grɑmɚ/	/dob/<br>/kinɪt/	/bɪnɪp/<br>/dob/<br>/grɑmɚ/<br>/sibl̩/
18J0573			/plun/<br>/topɪn/	/dob/<br>/grɑmɚ/<br>/plun/	/dob/<br>/grɑmɚ/<br>/plun/
18J0574	/topɪn/	/topɪn/	/grɑmɚ/<br>/nedɪg/<br>/plun/<br>/topɪn/		
19J0588			/plun/<br>/topɪn/		
19J0589			/plun/	/dob/<br>/plun/	/dob/
19J0593	/bɪnɪp/<br>/grɑmɚ/<br>/nedɪg/	/dob/<br>/grɑmɚ/<br>/kinɪt/<br>/mep/<br>/nedɪg/<br>/sibl̩/	/bɪnɪp/<br>/dob/<br>/grɑmɚ/<br>/mep/<br>/nedɪg/<br>/plun/<br>/sibl̩/		
19J0595	/dob/<br>/grɑmɚ/<br>/mep/<br>/nedɪg/<br>/sibl̩/	/dob/<br>/grɑmɚ/<br>/mep/<br>/nedɪg/<br>/sibl̩/<br>/topɪn/			
19J0597	/dob/<br>/nedɪg/	/plun/	/dob/<br>/plun/<br>/topɪn/	/dob/<br>/grɑmɚ/<br>/mep/<br>/plun/<br>/sibl̩/<br>/topɪn/	
19J0598	/nedɪg/	/nedɪg/	/nedɪg/<br>/sibl̩/	/grɑmɚ/<br>/nedɪg/	/grɑmɚ/<br>/nedɪg/<br>/sibl̩/<br>/topɪn/
19J0599	/dob/	/dob/	/dob/<br>/nedɪg/<br>/sibl̩/	/dob/<br>/nedɪg/<br>/sibl̩/	/dob/<br>/nedɪg/<br>/plun/<br>/sibl̩/
19J0601	/sibl̩/	/bɪnɪp/<br>/grɑmɚ/	/dob/<br>/grɑmɚ/	/dob/<br>/grɑmɚ/	/dob/<br>/grɑmɚ/

		/plun/<br>/sibḷ/	/nedɪg/<br>/sibḷ/	/kinɪt/<br>/mep/<br>/nedɪg/<br>/sibḷ/<br>/topɪn/	/kinɪt/<br>/mep/<br>/nedɪg/<br>/plun/<br>/sibḷ/<br>/topɪn/
19J0602	/mep/	/mep/	/dob/<br>/kinɪt/<br>/mep/<br>/nedɪg/<br>/plun/		
19J0604	/dob/	/dob/<br>/mep/<br>/nedɪg/<br>/sibḷ/			
19J0605	/dob/	/dob/<br>/plun/	/dob/<br>/plun/<br>/sibḷ/<br>/topɪn/	/dob/<br>/plun/<br>/sibḷ/<br>/topɪn/	

*18J0478 incorrectly said /plum/ for the target form /plun/ at beginning of session Free Recall on Days 3 through 6. However, the participant was not re-trained on this form during any of those sessions.
**18J0518 correctly said target forms /mep/ and /topɪn/ at beginning of session Free Recall on Day 5. The examiner retrained the forms in error.
***18J0542 correctly said target form /nedɪg/ at beginning of session Free Recall on Day 5. The examiner retrained the form in error.
