# Supplementary material for: Word Learning by Preschool-Age Children: Differences in Encoding, Re-Encoding, and Consolidation Across Learners During Slow Mapping
Source: J Speech Lang Hear Res. 2022 Apr 20;65(5):1956–77. doi: 10.1044/2022_JSLHR-21-00530 (PMC9559666; doi:10.1044/2022_JSLHR-21-00530)
Supplement: Supplemental Material S3 [file JSLHR-65-1956-s003.pdf]

Supplemental Material S3. Performance of individual children including the number of words correct at the last training day, and number of words trained to criterion.

Table S3.1. Number of words correct at the end of the last training day out of 9 trained forms. Only words produced with 100% phonological precision are included, with an exception for liquid simplification productions made for /grɑmɚ/. See Supplemental Material S1.

	**Total Training Days**	**Words Correct Free Recall**	**Words Correct Cued Recall**	**Words Demonstrated Criterion**
16C0368	5	3	8	8
16C0377	4	7	9	9
16C0409	5	6	9	8
17C0406	6	6	7	7
17C0426	5	5	9	9
17J0453	6	9	9	9
17J0455	6	6	6	6
18J0475	5	7	9	9
18J0478	6	5	5	5
18J0479	4	8	9	9
18J0482	2	9	9	9
18J0487	5	1	9	9
18J0488	3	9	9	9
18J0518	5	8	8	8
18J0519	6	4	8	7
18J0522	3	8	9	9
18J0538	6	6	9	9
18J0539	4	8	9	9
18J0540	6	0	5	5
18J0541	3	8	9	9
18J0542	6	8	8	8
18J0551	6	2	4	4
18J0555	3	8	9	9
18J0556	2	5	9	9
18J0560	6	6	7	7
18J0565	6	4	9	9
18J0567	4	5	9	9
18J0568	6	6	8	8
18J0571	6	7	8	8
18J0572	6	5	7	7
18J0573	6	1	8	6
18J0574	4	7	9	9
19J0588	4	6	9	9
19J0589	6	1	1	1
19J0593	4	8	9	9

19J0595	3	7	9	9
19J0597	5	7	9	9
19J0598	6	4	7	7
19J0599	6	5	7	7
19J0601	6	6	9	9
19J0602	4	4	8	8
19J0604	3	8	9	9
19J0605	5	4	9	9
Mean (standard deviation)	4.84 (1.27)	5.74 (2.33)	8.02 (1.67)	7.93 (1.70)
