# Supplementary material for: Word Learning by Preschool-Age Children: Differences in Encoding, Re-Encoding, and Consolidation Across Learners During Slow Mapping
Source: J Speech Lang Hear Res. 2022 Apr 20;65(5):1956–77. doi: 10.1044/2022_JSLHR-21-00530 (PMC9559666; doi:10.1044/2022_JSLHR-21-00530)
Supplement: Supplemental Material S4 [file JSLHR-65-1956-s004.pdf]

Supplemental Material S4. Specific characteristics of training days including average number of word trained each day and dosage of words within and across training days.

Table S4.1. Average number of words trained each day. Standard deviations in paratheses.

	Session 1	Session 2	Session 3	Session 4	Session 5	Session 6	Overall Average
Number of Words Trained	9*	7.58 (1.47)	6.37 (2.31)	5.34 (1.95)	4.44 (1.95)	4.42 (2.50)	5.93 (2.32)

*During session 1, all children were trained on all 9 words.

Table S4.2. Average number of words cued at the end of each session. Standard deviations in parenthesis.

	Session 1	Session 2	Session 3	Session 4	Session 5	Session 6	Overall Average
Number of Words Cued	7.26 (1.59)	5.47 (2.39)	4.32 (2.55)	3.91 (1.98)	4.30 (2.07)	3.95 (2.55)	5.06 (2.30)

Table S4.3. Number of exposures to the target form when target object was present. Numbers are based on whether a form-object pair was included in all training and testing blocks (trained that day), or only included in testing blocks (not trained that day). During each session, children received up to 3 additional exposures to the form as an alternate form in AFC trials. More details below.

	Session 1	All other training sessions
Form-object pair trained that day	12 exposures	9 exposures
Form-object pair not trained that day	—	3 exposures

During Session 1, children heard each form 12 times when the target object was present. They heard each form an additional 3 times as an alternate form in AFC tasks when another target object was present. Thus, total exposures to each form during Session 1 included 15 exposures. During subsequent training sessions, when a form-object was trained children heard each form 9 times when the object was present. When a form-object pair was not trained children heard the form 3 times, during the testing blocks, when the object was present. For both trained and untrained forms, the form was presented an additional time as an alternate form in the 4AFC task at the end of session. Each form could be presented an additional 2 times as an alternate form in 2AFC training blocks. However, this depended on which trials were eliminated due to a specific target form not being trained that day. For example, during Session 2 the first 2AFC training block included /plun/ as the target form and /sibl̩/ as the alternate form during one of the trials. If the child correctly produced /plun/ at the beginning of that training session, then this trial was not administered. The second 2AFC training block, included /bɪnɪp/ as the target form and /sibl̩/ as an alternate form during one of the trials. If the child correctly produced /bɪnɪp/ at the beginning

of that training session, then this training trial was not administered. Thus, /sibl̩/ could be presented as an alternate form 0, 1, or 2 times during the 2AFC blocks based on how the child performed at the beginning of that training session. Total exposures to each form during Session 2 (and all subsequent training sessions) included between 3 to 5 exposures if the word was not trained that session, and 10 to 12 exposures if the word was trained that session.
