# Supplementary material for: Word Learning by Preschool-Age Children: Differences in Encoding, Re-Encoding, and Consolidation Across Learners During Slow Mapping
Source: J Speech Lang Hear Res. 2022 Apr 20;65(5):1956–77. doi: 10.1044/2022_JSLHR-21-00530 (PMC9559666; doi:10.1044/2022_JSLHR-21-00530)
Supplement: Supplemental Material S5 [file JSLHR-65-1956-s005.pdf]

Supplemental Material S5. Final models for all analyses with PPVT scores and final models for all analyses with age in months.

Table S5.1. Final models for word learning performance across training days, whole word score with PPVT.

**Log Odds of Producing a Word Correctly**				
	**Estimate**	**Standard Error**	***z* value**	**Pr(>|*z*|)**
Intercept	–1.43	0.33	–4.35	< 0.0001
Day[a]	0.62	0.05	11.40	< 0.0001
Time[b]	0.73	0.09	7.98	< 0.0001
PPVT	0.03	0.01	2.31	0.02
PPVT × Time	0.003	0.01	0.42	0.67
Biological Sex[c]	–0.004	0.38	–0.01	0.99
Maternal Ed	0.04	0.10	0.36	0.72

[a]Day 2 was coded as 0 to reflect performance at the intercept. [b]Beginning of session was coded as –.05. End of session was coded as .05. [c]Female was coded as –.05. Male was coded as .05.

Table S5.2. Final models for word learning performance across training days, phonological precision score with PPVT.

**Percentage of Phonetic Features Produced correctly**				
	**Estimate**	**Standard Error**	***t* value**	**Pr(>|*z*|)**
Intercept	0.34	0.06	5.62	< 0.0001
Day[a]	0.11	0.01	11.97	< 0.0001
Time[b]	0.12	0.02	7.82	< 0.0001
PPVT	0.01	0.002	1.92	0.06
PPVT × Time	0.0001	0.001	0.10	0.92
Biological Sex[c]	0.03	0.06	0.50	0.62
Maternal Ed	0.02	0.02	0.96	0.34

[a]Day 2 was coded as 0 to reflect performance at the intercept. [b]Beginning of session was coded as –.05. End of session was coded as .05. [c]Female was coded as –.05. Male was coded as .05.

Table S5.3. Final models for consolidation during training, whole word score with PPVT.

**Log Odds of Producing a Word Correctly**				
	**Estimate**	**Standard Error**	***z* value**	**Pr(>|*z*|)**
Intercept	–2.05	0.33	–6.18	< 0.0001
Gap[a]	0.71	0.04	16.47	< 0.0001
Time[b]	–0.01	0.09	–0.15	0.88
PPVT	0.04	0.01	2.70	< 0.01
PPVT × Time	–0.003	0.01	–0.42	0.67
Biological Sex[c]	–0.07	0.35	–0.189	0.86
Maternal Ed	0.03	0.09	0.31	0.75

[a]Gap 1 was coded as 0 to reflect performance at the intercept. [b]Beginning of session was coded as –.05. End of session was coded as .05. [c]Female was coded as –.05. Male was coded as .05.

Table S5.4. Final models for consolidation during training, phonological precision score with PPVT.

**Percentage of Phonetic Features Produced correctly**				
	**Estimate**	**Standard Error**	***t* value**	**Pr(>|*z*|)**
Intercept	0.26	0.05	5.17	< 0.001
Gap[a]	0.12	0.01	10.32	< 0.0001
Time[b]	0.003	0.02	0.22	0.83
PPVT	0.002	0.002	1.14	0.26
PPVT × Time	–0.001	0.001	–0.91	0.36
Biological Sex[c]	0.03	0.05	0.52	0.60
Maternal Ed	0.01	0.01	0.99	0.33

[a]Gap 1 was coded as 0 to reflect performance at the intercept. [b]Beginning of session was coded as –.05. End of session was coded as .05. [c]Female was coded as –.05. Male was coded as .05.

Table S5.5. Final models assessing the effect of delay (end of training, one-month delay) on production of words, whole word score with PPVT.

**Log Odds of Producing a Word Correctly**				
	**Estimate**	**Standard Error**	***z* value**	**Pr(>|*z*|)**
Intercept	0.18	0.31	0.59	0.56
Time[a]	–0.66	0.17	–3.93	< 0.0001
PPVT	0.03	0.01	1.94	0.05
PPVT × Time	0.0005	0.01	0.04	0.97
Biological Sex[b]	–0.12	0.34	–0.34	0.73
Condition, Contrast 1[c]	–0.10	0.21	–0.48	0.63
Condition, Contrast 2[d]	0.16	0.12	1.39	0.17
Condition × Time, Contrast 1	0.33	0.21	1.61	0.11
Condition × Time, Contrast 2	–0.07	0.12	–0.56	0.58
Maternal Ed	0.09	0.09	1.00	0.32

[a]End of last training session was coded as –.05. Beginning of one-month session was coded as .05. [b]Female was coded as –.05. Male was coded as .05. [c]Retest at one week was coded as –1, Retest at two weeks was coded as 1, No retest was coded as 0. [d]Retest at one week was coded as –1, Retest at two weeks was coded as –1, No retest was coded as 2.

Table S5.6. Final models assessing the effect of delay (end of training, one-month delay) on production of words, phonological precision score with PPVT.

**Percentage of Phonetic Features Produced correctly**				
	**Estimate**	**Standard Error**	***t* value**	**Pr(>|*z*|)**
Intercept	0.59	0.06	9.39	< 0.0001
Time[a]	–0.13	0.03	–4.40	< 0.0001
PPVT	0.003	0.003	1.39	0.17
PPVT × Time	0.001	0.002	0.47	0.64
Biological Sex[b]	0.02	0.07	0.25	0.81
Condition, Contrast 1[c]	–0.03	0.04	–0.60	0.55
Condition, Contrast 2[d]	0.03	0.02	1.20	0.24
Condition × Time, Contrast 1	0.08	0.04	2.30	0.02
Condition × Time, Contrast 2	0.001	0.02	0.05	0.96
Maternal Ed	0.02	0.02	1.18	0.24

[a]End of last training session was coded as –.05. Beginning of one-month session was coded as .05. [b]Female was coded as –.05. Male was coded as .05. [c]Retest at one week was coded as –1, Retest at two weeks was coded as 1, No retest was coded as 0. [d]Retest at one week was coded as –1, Retest at two weeks was coded as –1, No retest was coded as 2.

Table S5.7. Final models for word learning performance across training days, whole word score with age in months.

**Log Odds of Producing a Word Correctly**				
	**Estimate**	**Standard Error**	***z* value**	**Pr(>|*z*|)**
Intercept	–1.42	0.34	–4.21	< 0.0001
Day[a]	0.58	0.04	14.43	< 0.0001
Time[b]	0.73	0.09	7.98	< 0.0001
Age in months	0.04	0.02	1.81	0.07
Age × Time	0.01	0.01	1.39	0.16
Biological Sex[c]	0.17	0.39	0.43	0.67
Maternal Ed	0.09	0.11	0.83	0.40

[a]Day 2 was coded as 0 to reflect performance at the intercept. [b]Beginning of session was coded as –.05. End of session was coded as .05. [c]Female was coded as –.05. Male was coded as .05.

Table S5.8. Final models for word learning performance across training days, phonological precision score with age in months.

**Percentage of Phonetic Features Produced correctly**				
	**Estimate**	**Standard Error**	***t* value**	**Pr(>|*z*|)**
Intercept	0.34	0.06	5.59	< 0.0001
Day[a]	0.11	0.01	12.00	< 0.0001
Time[b]	0.12	0.01	7.90	< 0.0001
Age in months	0.01	0.004	1.53	0.13
Age × Time	0.001	0.002	0.77	0.44
Biological Sex[c]	0.07	0.06	1.04	0.30
Maternal Ed	0.02	0.02	1.30	0.20

[a]Day 2 was coded as 0 to reflect performance at the intercept. [b]Beginning of session was coded as –.05. End of session was coded as .05. [c]Female was coded as –.05. Male was coded as .05.

Table S5.9. Final models for consolidation during training, whole word score with age in months.

**Log Odds of Producing a Word Correctly**				
	**Estimate**	**Standard Error**	***z* value**	**Pr(>|*z*|)**
Intercept	–2.05	0.34	–6.09	< 0.0001
Gap[a]	0.71	0.04	16.44	< 0.0001
Time[b]	–0.01	0.09	–0.12	0.90
Age in months	0.04	0.02	1.81	0.07
Age × Time	0.01	0.01	1.03	0.30
Biological Sex[c]	0.20	0.37	0.54	0.59
Maternal education	0.08	0.10	0.77	0.44

[a]Gap 1 was coded as 0 to reflect performance at the intercept. [b]Beginning of session was coded as –.05. End of session was coded as .05. [c]Female was coded as –.05. Male was coded as .05.

Table S5.10. Final models for consolidation during training, phonological precision score with age in months.

**Percentage of Phonetic Features Produced correctly**				
	**Estimate**	**Standard Error**	***t* value**	**Pr(>|*z*|)**
Intercept	0.26	0.05	5.20	< 0.001
Gap[a]	0.12	0.01	10.39	< 0.0001
Time[b]	0.005	0.01	0.37	0.71
Age in months	0.005	0.003	1.79	0.08
Age × Time	0.001	0.002	0.69	0.49
Biological Sex[c]	0.05	0.05	1.01	0.32
Maternal Ed	0.02	0.01	1.45	0.16

[a]Gap 1 was coded as 0 to reflect performance at the intercept. [b]Beginning of session was coded as –.05. End of session was coded as .05. [c]Female was coded as –.05. Male was coded as .05.

Table S5.11. Final models assessing the effect of delay (end of training, one-month delay) on production of words, whole word score with age in months.

**Log Odds of Producing a Word Correctly**				
	**Estimate**	**Standard Error**	***z* value**	**Pr(>|*z*|)**
Intercept	0.49	0.47	1.03	0.30
Time[a]	–0.94	0.30	–3.17	< 0.001
Age in months	0.04	0.02	1.80	0.07
Age × Time	–0.03	0.02	–1.72	0.09
Biological Sex[b]	0.08	0.35	0.23	0.82
Condition, Contrast 1[c]	–0.38	0.48	–0.79	0.43
Condition, Contrast 2[d]	0.36	0.47	0.76	0.45
Condition × Time, Contrast 1	0.17	0.41	0.42	0.68
Condition × Time, Contrast 2	–0.07	0.12	–0.56	0.58
Maternal Ed	0.12	0.10	1.26	0.21

[a]End of last training session was coded as –.05. Beginning of one-month session was coded as .05. [b]Female was coded as –.05. Male was coded as .05. [c]Retest at one week was coded as –1, Retest at two weeks was coded as 1, No retest was coded as 0. [d]Retest at one week was coded as –1, Retest at two weeks was coded as –1, No retest was coded as 2.

Table S5.12. Final models assessing the effect of delay (end of training, one-month delay) on production of words, phonological precision score with age in months.

**Percentage of Phonetic Features Produced correctly**				
	**Estimate**	**Standard Error**	***t* value**	**Pr(>|*z*|)**
Intercept	0.59	0.06	9.35	< 0.0001
Time[a]	–0.13	0.03	–4.40	< 0.0001
Age in months	0.003	0.004	0.89	0.38
Age × Time	–0.0–0.4	0.003	–1.15	0.25
Biological Sex[b]	0.04	0.07	0.64	0.52
Condition, Contrast 1[c]	–0.01	0.04	–0.34	0.74
Condition, Contrast 2[d]	0.03	0.02	1.13	0.26
Condition × Time, Contrast 1	0.08	0.04	2.30	0.02
Condition × Time, Contrast 2	0.002	0.02	0.10	0.92
Maternal Ed	0.03	0.02	1.36	0.18

[a]End of last training session was coded as –.05. Beginning of one-month session was coded as .05. [b]Female was coded as –.05. Male was coded as .05. [c]Retest at one week was coded as –1, Retest at two weeks was coded as 1, No retest was coded as 0. [d]Retest at one week was coded as –1, Retest at two weeks was coded as –1, No retest was coded as 2.
