# Supplementary material for: Word Learning by Preschool-Age Children: Differences in Encoding, Re-Encoding, and Consolidation Across Learners During Slow Mapping
Source: J Speech Lang Hear Res. 2022 Apr 20;65(5):1956–77. doi: 10.1044/2022_JSLHR-21-00530 (PMC9559666; doi:10.1044/2022_JSLHR-21-00530)
Supplement: Supplemental Material S6 [file JSLHR-65-1956-s006.pdf]

Supplemental Material S6. Effect of retest on performance at one month.

**Mean Number of Words Produced Correctly**				
	**End of Training**	**1 week retest**	**2 weeks retest**	**1 Month**
Condition 1, Retest at 1 week	5.50 (2.35)	5.00 (2.11)		3.93 (2.13)
Condition 2, Retest at 2 weeks	4.71 (2.49)		4.43 (1.99)	4.29 (2.16)
Condition 3, No retest	6.00 (2.10)			4.67 (1.76)
Average Across Conditions	5.42 (2.32)			4.30 (1.99)
**Mean Percentage of Phonemic Features Produced Correctly**				
	**End of Training**	**1 week retest**	**2 weeks retest**	**1 month**
Condition 1, Retest at 1 week	0.70 (0.45)	0.63 (0.47)		0.48 (0.49)
Condition 2, Retest at 2 weeks	0.57 (0.49)		0.56 (0.48)	0.53 (0.49)
Condition 3, No retest	0.70 (0.45)			0.58 (0.49)
Average Across Conditions	0.62 (0.47)			0.56 (0.48)

Children were semi-randomly assigned to receive retesting one week (n = 14), two weeks (n = 14) or no retest (n = 15) before the one-month session. Semi-random assignment was used to retain comparable biological sex, ages, and PPVT scores across groups. There was some variation in when children completed these long-term sessions. For the one-week condition, children's memory was assessed between 5 and 9 (mean = 6.78) days after the last training session; for the two-week condition, children's memory was assessed between 13 and 15 (mean = 13.64) days after the last training session.

Children in the three conditions did not differ in the number of words produced after the one-month delay. Additionally, there was not an overall effect of retest in the phonological precision of productions. Children in the no retest group produced a similar phonological precision of productions at the 1-month delay as children in the retest groups (B = 0.0007, t = 0.04, p = .97). However, children who were retested in the two-week condition produced forms with more phonological precision at the one-month test than children who were retested in the one-week condition (B = 0.09, t = 2.36, $p < .05$). Current research with adults demonstrates that depending on when the final test is administered, such as one-month after training, there is a sweet-spot at which the words should be retested to lead to optimal performance (Cepeda, Pashler, Vul, Wixted, & Rohrer, 2006). However, results in the current study are inconclusive about whether the retest was helpful and when the retest should be administered. Notably, when we administered the retest protocol we did provide feedback in the last training block but we did

not offer retraining on target word-referent pairs. Through further research, we can identify whether retesting and retraining improve long-term retention. Overall, the supportive initial training appears to foster long-term retention. Children who vary in language skills demonstrated fairly good retention of trained words when tested one-month later.
